# Carbon Quantum Dots Derived from Garlic (*Allium sativum*) Peel as Corrosion Inhibitor for Mild Steel in HCl Solution

**DOI:** 10.1021/acsomega.5c03224

**Published:** 2025-07-22

**Authors:** Gabriel Kajiyama Kuriya, Victor Magno Paiva, Sanair Massafra de Oliveira, Agnes Candido Teixeira, Clara Muniz da Silva de Almeida, Jairo Eduardo Leiva Mateus, Joyce Rodrigues de Araujo, Marcelo Eduardo Huguenin Maia da Costa, Natasha Midori Suguihiro, Eliane D’Elia

**Affiliations:** 1 Department of Inorganic Chemistry, 28125 Universidade Federal do Rio de Janeiro, Avenida Athos da Silveira Ramos, 149, Cidade Universitária, Rio de Janeiro 21941-909, Brazil; 2 Materials Metrology Division, Instituto Nacional de Metrologia, 119536 Qualidade e Tecnologia INMETRO Avenida Nossa Sra. das Graças, 50, Xerém, Duque de Caxias 25250-020, Brazil; 3 Nanotechnology Engineering Program, 28125 Universidade Federal do Rio de Janeiro, Avenida Horácio Macedo 2030 Cidade Universitária, Rio de Janeiro 21941-972, Brazil; 4 Department of Mechanical Engineering, 28099 Pontifícia Universidade Católica do Rio de Janeiro, Rua Marquês de São Vicente, 225, Gávea, Rio de Janeiro 22451-900, Brazil; 5 Department of Physics Pontifícia Universidade Católica do Rio de Janeiro, Rua Marquês de São Vicente, 225, Gávea, Rio de Janeiro 22451-900, Brazil; 6 Department of Nanotecnology, Universidade Federal do Rio de Janeiro Campus UFRJ, Duque de Caxias Professor Geraldo Cidade, Rodovia Washington Luiz 19593, Duque de Caxias 25240-005, Brazil

## Abstract

In this study, carbon quantum dots (CQDs) were successfully synthesized from garlic peel, with size dimensions confirmed by TEM and DLS analyses and photochemical properties validated by UV–vis and fluorescence spectroscopy. FTIR revealed chemical similarities with the precursor, while XPS revealed the presence of nitrogen and sulfur, indicating endogenous doping. The anticorrosive performance of the CQDs was demonstrated through gravimetric, electrochemical impedance, potentiodynamic polarization, and surface analyses including scanning electron microscopy, atomic force microscopy, and X-ray photoelectron spectroscopy. The CQDs exhibited high inhibition efficiencies with sustained performance over time, achieving maximum inhibition efficiencies of 79% for 2 h and 96% for 24 h, as determined by gravimetric studies. This inhibition was primarily mediated through physical interactions, as indicated by temperature-dependent studies. Electrochemical measurements further confirm that the CQDs act as mixed-type inhibitors, exhibiting a predominant anodic effect. Surface analyses confirmed the formation of a protective and hydrophobic film on the steel surface. XPS, DLS, and zeta potential studies, along with Arrhenius equation analysis, demonstrated that the film forms through physical and chemical interactions between mild steel and quantum dot aggregates. These findings highlight the potential of using agro-industrial waste, such as garlic peel, as a sustainable precursor for the synthesis of effective corrosion inhibitors.

## Graphical abstract

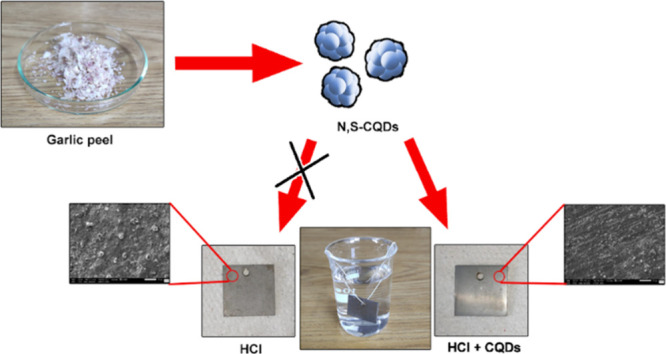

## 1 Highlights

1. Synthesized carbon quantum dots (CQDs) from garlic peel using acid hydrolysis.
2. Achieved up to 79 and 96% inhibition efficiency for mild steel in 1 mol L^–1^ HCl after 2 and 24 h, respectively.
3. The inhibition mechanism involves both physical and chemical interactions between CQD aggregates and the steel surface.
4. Surface analysis revealed hydrophobic protective film formation by CQDs on steel, reducing metal dissolution.
5. Presents a sustainable and potential use of agro-waste to produce carbon quantum dots and effective inhibitors of corrosion.

## 2 Introduction

Mild steel (MS) is one of the most widely used materials in the industry due to its low cost, mechanical properties, the possibility of recycling, and machinability without losing its properties.^1,2^ Therefore, it is widely used in the industry, especially in the oil industry. MS is the standard pipeline material that transports oil, gas, water, and other fluids.^3^ The transportation of such products favors corrosion and incrustation (formation of oxides), which slows or prevents the passage of fluids.^3^

One way to avoid this problem is to acid pickle MS to remove oxides.^1,2,4^ Hydrochloric acid is usually used in this method due to its low cost and ability to form water-soluble metal chlorides.^1,2,4^ Despite the procedure’s effectiveness, the acid corrodes the steel, causing damage to industrial pipelines.^1,2,4^ Therefore, the industry makes extensive expenditures for repairing and replacing pipes.^3–5^

Organic corrosion inhibitors are widely used to minimize metal degradation. These compounds act by adsorbing onto the metal surface, forming a protective layer that prevents direct contact with the corrosive medium, protecting the active corrosion sites.^5,6^ These molecules are present in their structure compounds such as N, S, P, and/or O that allow adsorption onto the carbon steel surface by physical and/or chemical interactions.^6^ Other characteristics, such as multiple bonds and aromatic rings, also increase interactions.^4,5^

Thus, carbon quantum dots (CQDs), which are nanoparticles smaller than 10 nm, are excellent candidates as corrosion inhibitors due to their potential richness in functional groups—depending on the synthesis method and starting materials—which allows for easy modification of their chemical structure.

In recent years, certain research groups have taken a green approach by developing methodologies for synthesizing CQDs derived from biomass residues.^9–12^ The use of raw materials is favorable to the environment and the inhibitory efficiency of the synthesized CQDs, as the organic residue is rich in O, N, P, and S.^8–12^ With the increase in population, food production grows exponentially, resulting in the production of large amounts of agro-industrial waste that often goes untreated, thereby increasing environmental pollution.^13^

Recent literature has highlighted various biomass sources for CQD synthesis with effective corrosion inhibition properties. For example, pitaya and grapefruit peels have yielded CQDs with inhibition efficiencies above 94% in hydrochloric acid environments, demonstrating their assertive protective behavior against corrosive attacks on mild steel surfaces.^14,15^ Zheng et al. reported that CQDs synthesized from biomass waste can self-assemble on metal surfaces, forming uniform and adherent protective films that hinder electrolyte access and reduce corrosion rates.^15^ Long et al. and Kamaruzzaman et al. further advanced the field by developing CQDs doped with nitrogen and sulfur, demonstrating improved corrosion resistance due to enhanced surface interactions with the metal substrate, which confirmed the role of heteroatom doping in the inhibitor performance.^16,17^ Furthermore, recent studies by Zamindar et al. and Verma et al. have emphasized green synthesis approaches for CQDs, highlighting the importance of using environmentally benign precursors while achieving high corrosion inhibition efficiencies, thereby aligning with global sustainability goals.^18,19^ These advancements demonstrate the versatility of CQDs from various biomass sources and reinforce the ongoing trend toward ecofriendly and high-performance corrosion inhibitors.

Despite the advances, few studies have thoroughly investigated garlic peel, a high-sulfur agricultural waste, as a precursor for CQDs for corrosion protection. Garlic (*Allium sativum*) is used in many cultures worldwide as a food seasoning and has been cultivated on a large scale for centuries.^20,21^ Garlic peel is generated as waste during garlic processing and contributes to environmental pollution, like most agro-industrial waste.^13,20–22^ Approximately 3.3 billion tons of CO_2_ are generated from untreated agro-industrial waste each year, which increases the greenhouse effect and, consequently, global warming.^13^ Part of the CO_2_ released comes from burning waste from garlic production, which amounts to 3.5 million tons annually.^22^

A range of bioactive compounds is found in garlic bulbs and skin, primarily aromatic compounds and organosulfur compounds, including flavonoids, polyphenols, allicin, and alliin, as well as polysaccharides, which can facilitate the incorporation of several functional groups into the structure of CQDs.^20^ Such compositional characteristics and the high carbon content of garlic skin are desirable in CQDs, making the residue an excellent candidate as a precursor for synthesizing carbon quantum dots with compositional sulfur.

Therefore, this work aimed to produce CQDs from garlic (*A. sativum*) peel and their application as a green corrosion inhibitor for mild steel in a 1 mol L^–1^ HCl solution, a low-carbon technology that can reduce part of the burning and pollution associated with waste from garlic production.

## 3 Materials and Methods

### 3.1 Carbon Quantum Dot Synthesis

For the synthesis of carbon quantum dots, 1 g of previously crushed garlic peel (*A. sativum*) was added to a round-bottom flask with 20 mL of 1 mol L^–1^ sulfuric acid solution prepared from 98% sulfuric acid (purchased from ISOFAR). The mixture was heated under reflux in a heating mantle at 200 °C for 2 h and then filtered through a 0.45 μm pore-size membrane to remove residues of garlic peel and large particles. The filtered solution was dialyzed for 48 h using a 1 kDa membrane with a capacity of 20 mL (PUR-A-LYZER) to remove smaller residues, with water exchange twice daily. Subsequently, vacuum filtration filtered the solution through a 0.22 μm pore membrane (KASVI) to remove the remaining solid residues. Afterward, the remaining solution was dried with a rotary evaporator. The dried material was resuspended as a stock solution of 1 mg mL^–1^. This method yielded approximately 10% of the produced CQD. A simplified diagram is shown in Figure 1.

**Figure 1:**
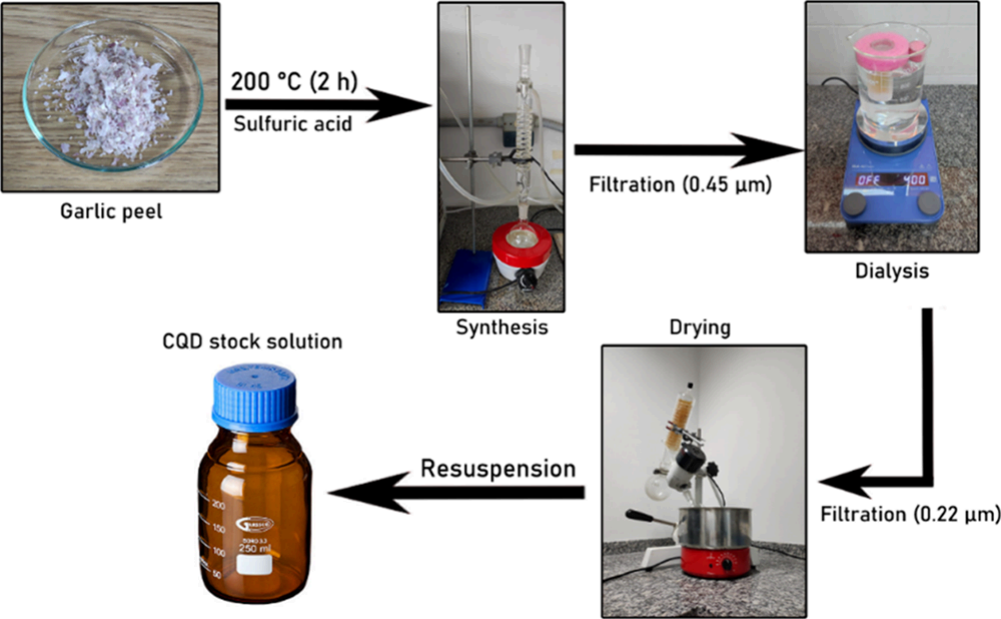
Simplified diagram of CQD synthesis and purification from garlic peel.

### 3.2 Characterization

The morphological characteristics, chemical composition, and optical properties of quantum dots were analyzed by using several techniques, such as transmission electron microscopy (TEM), dynamic light scattering (DLS), Fourier transform infrared (FTIR) spectroscopy, Raman spectroscopy, X-ray photoelectron spectroscopy (XPS), Zeta potential measurement, UV–vis spectroscopy, and fluorimetry.

TEM investigation was performed by dropping the carbon quantum dot (CQD) solution onto a lacey carbon-coated copper grid (Cu grid). The characterization was carried out by using a JEOL JEM-2100 Plus transmission electron microscope, operating at an accelerating voltage of 200 kV.

DLS measurements were conducted with an Anton Paar Litesizer 500, utilizing a 0.2 mg mL^–1^ dispersion of CQDs in DMF. FTIR analysis was performed on a Nicolet 6700 FTIR spectrometer, with samples prepared in KBr and spectra recorded in the 400 to 4000 cm^–1^ range. X-ray photoelectron spectroscopy (XPS) was performed using an ultrahigh vacuum system (ScientaOmicron) with a nonmonochromatic Al Kα X-ray source (hν = 1486.6 eV), operating at 15 kV and 20 mA. The survey scan covered an energy range from 0 to 1100 eV, while high-resolution spectra were obtained based on the sample composition at an energy setting of 30 eV.

Raman spectra were collected using a Confocal Raman Microscope (SENTERRA II) with a 532 nm laser excitation. The UV–vis spectrum was measured from 200 to 700 nm with a Shimadzu UV-1800 spectrophotometer, and fluorescence measurements were taken by using a Shimadzu RF 5301 cuvette fluorimeter. Fluorometric analysis employed excitation wavelengths in increments of 300 to 500 nm. For these experiments, a 0.2 mg mL^–1^ suspension was used for the UV–vis analysis, and a 1 mg mL^–1^ suspension was used for the fluorescence spectroscopy.

Lastly, the stability of the particles was assessed through Zeta potential measurements using an Anton Paar Litesizer 500. Samples at a concentration of 1 mg mL^–1^ in Milli-Q water underwent 60 runs at a potential of 200 V and a temperature of 25 °C.

### 3.3 Mild Steel Surface Preparation

The corrosion tests utilized mil steel with the following composition: carbon (C), 0.18%; manganese (Mn), 0.30%; phosphorus (P), 0.04%; sulfur (S), 0.05%, with trace amounts of silicon (Si) and iron (Fe) making up the balance. Before the corrosion analyses were conducted by gravimetry, the mild steel plates, each with a surface area of approximately 13 cm^2^, were polished using sandpaper with grits of 100, 320, and 600 mesh. After abrading, the plates were thoroughly cleaned with deionized water. A smaller mild steel plate with a surface area of about 0.8 cm^2^ was employed for the electrochemical tests.

### 3.4 Gravimetric Tests

The mild steel plates were immersed in 1 mol L^–1^ hydrochloric acid prepared from 37% hydrochloric acid (purchased from Isofar) in the absence and presence of inhibitor for 2 and 24 h at room temperature. The inhibitor’s concentrations were 50, 100, 200, 300, and 400 ppm for the 2 h and 50, 100, 200, and 300 ppm for the 24 h. Before being immersed, the plates were weighed on an analytical balance, and their dimensions were measured with a caliper. The mass variation of the plates before and after each test was used to calculate the corrosion rate (eq 1) and the corrosion inhibition efficiency (eq 2).^23,24^

The effect of the temperature on the inhibitory process was analyzed by varying the temperature in gravimetric tests. Thus, immersion tests were conducted at temperatures of 25, 35, 45, and 55 °C for 2 h at a fixed inhibitor concentration of 200 ppm. eqs 1 and 2 also obtained the corrosion rate (*W*_corr_ in mg cm^–2^ h^–1^) and inhibition efficiency, respectively:

$$W_{\mathrm{corr}}=\frac{\Delta m}{tA}$$ 1

where Δ*m* represents the variation in sample mass (in mg), *t* the immersion time (in h), and *A* the exposed area of the sample (in cm^2^).

$$\mathrm{IE}\%=\frac{W_{\mathrm{corr},0}-W_{\mathrm{corr}}}{W_{\mathrm{corr},0}}$$ 2

*W*_(corr,0)_ and *W*_corr_ represent the corrosion rates for the medium without and with the inhibitor, respectively.

### 3.5 Electrochemical Tests

The electrochemical tests were performed in an electrochemical cell with a standard 3-electrode system consisting of a saturated calomel electrode (SCE) as the reference electrode, a platinum counter electrode, and a mild steel plate as the working electrode. The measurements were taken with an AUTOLAB potentiostat at room temperature. The data were obtained for 1 mol L^–1^ hydrochloric acid without and with the inhibitor at 50, 100, 200, and 300 ppm concentrations.

The electrode circuit potential (OCP) was measured for 2 h to verify the system’s stability. Then, the electrochemical impedance data were obtained with a 10 mV amplitude sine wave at 10^5^ to 10^–2^ Hz. Finally, polarization curves were acquired from −300 to +300 V vs OCP with a scan rate of 1 mV s^–1^.

The inhibition efficiency calculated by electrochemical impedance used the charge transfer resistance, *R*_ct_ values. The inhibition efficiency was obtained using eq 3 ^25^, represented below:

$$\mathrm{IE}\%=\frac{R_{\mathrm{ct}}-R_{\mathrm{ct},0}}{R_{\mathrm{ct}}}$$ 3

*R*_ct,0_ and *R*_ct_ represent the charge transfer resistance in the absence and presence of the inhibitor, respectively.

From the polarization, the IE% was obtained from eqs 4 and 5, where *j*_(corr,0)_ and *j*_corr_ represent the corrosion current density in the absence and presence of the inhibitor, respectively. In contrast, *R*_*p*_ and *R*_*p*,0_ represent the polarization resistance in the presence and absence of an inhibitor, respectively:

$$\mathrm{IE}\%=\frac{j_{\mathrm{corr},0}-j_{\mathrm{corr}}}{j_{\mathrm{corr},0}}$$ 4

$$\mathrm{IE}\%=\frac{R_{p}-R_{p,0}}{R_{p}}$$ 5

### 3.6 Surface Analysis

The effect of corrosive media on the surface of mild steel was assessed by using contact angle measurements, scanning electron microscopy (SEM), atomic force microscopy (AFM), and X-ray photoelectron spectroscopy (XPS).

Scanning electron microscopy images were acquired using a FEG-SEM JEOL JSM-IT700HR microscope, operated at 5 kV with secondary electron detection. To determine the hydrophobicity of the surfaces, contact angles were measured with an automatic tensiometer (Teclis Instrument, France) fitted with a 2.11 mm diameter aluminum needle. Measurements were performed at room temperature (20.3 °C) and 56% relative humidity. 2 μL of ultrapure water (resistivity 18.2 MΩ cm) was placed on each surface to evaluate the surface homogeneity. The needle and syringe were thoroughly cleaned before each measurement. For each sample, three independent measurements were taken at different spots. The contact angle was recorded once the droplet reached a stable shape, typically around 25 s after deposition. The final contact angle values were averaged to provide a representative result.

AFM imaging was conducted using a NanoWizard instrument from JPK, operating in intermittent contact mode with a silicon probe with a spring constant of 3 N m^–1^ and a resonance frequency of approximately 70 kHz. The root-mean-square (RMS) surface roughness was calculated for 30 mm × 30 mm images using the JPK software to estimate surface texture. The RMS values were derived from the average of 10 measurements.

X-ray photoelectron spectroscopy was conducted using an ultrahigh vacuum system (Specs System equipped with a Phoibos 150 Hemispherical analyzer) with a nonmonochromatic Al Kα X-ray source (hν = 1486.6 eV), operating at 15 kV and 20 mA. The survey scan covered an energy range from 0 to 1100 eV, while high-resolution spectra were obtained based on the sample composition at an energy setting of 30 eV.

## 4 Results and Discussion

### 4.1 Carbon Quantum Dot Characterization

One important physical property for characterizing carbon quantum dots (CQDs) is their size, typically less than 10 nm in diameter.^26^ Therefore, the nanomaterials produced were characterized by TEM and DLS. As shown in Figure 2A, the sample exhibits a narrow size distribution. Additionally, Figure 2B confirms that the synthesized particles are smaller than 10 nm. In both images, the absence of lattice fringes suggests that the material has an amorphous nature.^27^ From the TEM image, a histogram (Figure 2C) of the obtained nanomaterials was created, revealing that the carbon quantum dots were successfully produced and had an average size of 3.17 ± 0.07 nm.

**Figure 2:**
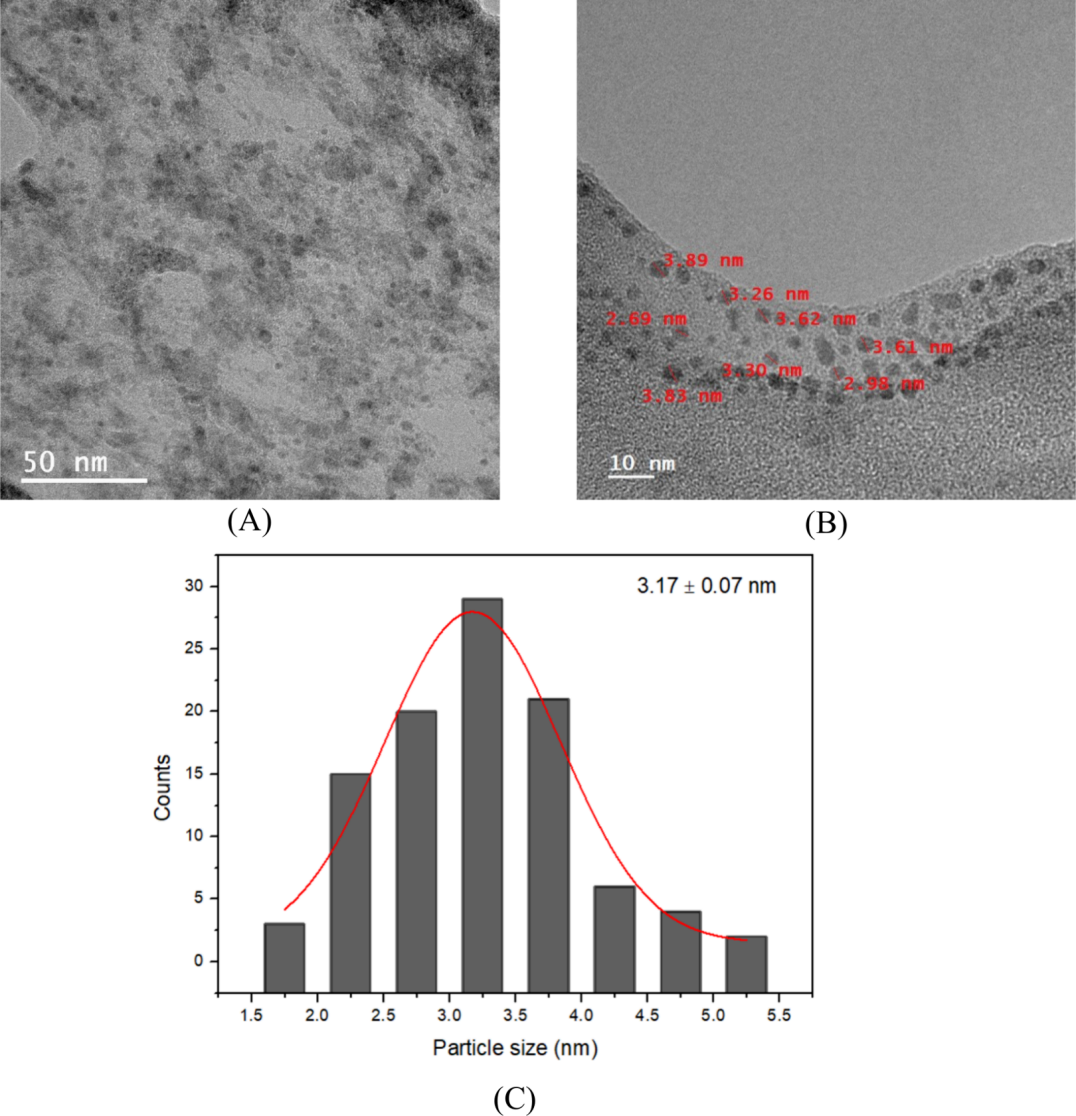
TEM images of carbon quantum dots (A,B) and a histogram of carbon quantum dots size (C).

Figure 3 illustrates the size distribution of the synthesized CQDs, showing an average hydrodynamic diameter of 2.29 nm and a polydispersity index of 0.24 in DMF, corroborating what was observed in the TEM analysis. These results show that PQCs were successfully produced.

**Figure 3:**
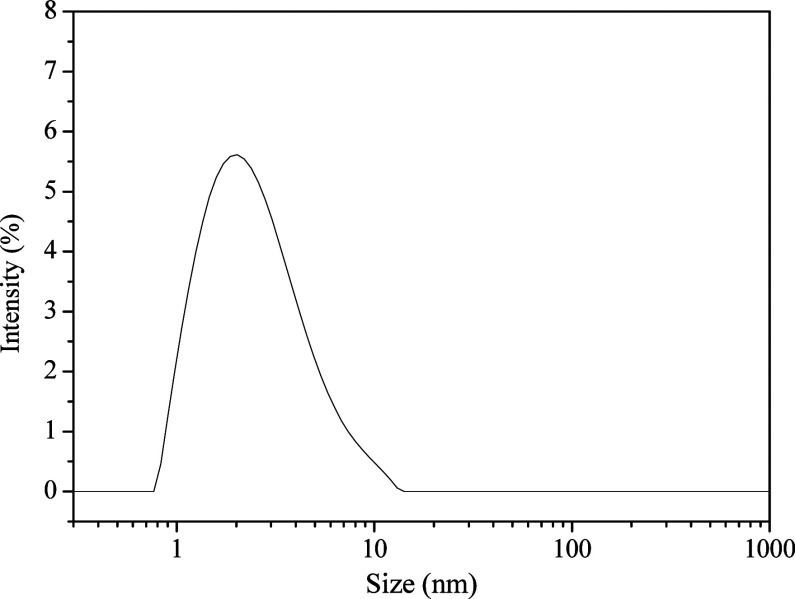
Dynamic light scattering (DLS) size distribution of CQDs obtained from garlic peel in the concentration of 0.02 mg L^–1^ in DMF.

In addition to having a size smaller than 10 nm, photoluminescence is another essential characteristic of carbon quantum dots (CQDs).^28^ Figure 4 illustrates the emission spectrum of CQDs synthesized at different excitation wavelengths. This figure highlights the photoluminescent behavior of the CQDs. Three distinct emission peaks are observed when the sample is excited with UV light at wavelengths of 300, 310, and 320 nm. These peaks indicate the presence of CQD particles with varying sizes, as shown in Figure 2, which produces different excitation peaks.^29,30^ Other excitation wavelengths also fail to show emission focused on a single peak, further suggesting diversity in nanoparticle sizes in aqueous media, thereby supporting the results obtained from dynamic light scattering (DLS). The highest emission intensity occurs at 440 nm when excited with UV light at a wavelength of 340 nm. As the UV excitation wavelength increases, the corresponding peak shifts to longer emission wavelengths, resulting in a consequent decrease in the intensity. The other emission peaks exhibit similar behavior, indicating that the synthesized CQDs have an irregular distribution of functional groups on their surfaces.^30–33^ Thus, it is possible to say that the emission wavelength of the produced CQD is directly dependent on the excitation wavelength of the UV light. Considering that the highest emission spectrum occurs at a high energy wavelength (340 nm), it is possible to say that its bandgap is also high, a characteristic only possible for smaller than 10 nm,^34^ which proves once again the synthesis of CQD.

**Figure 4:**
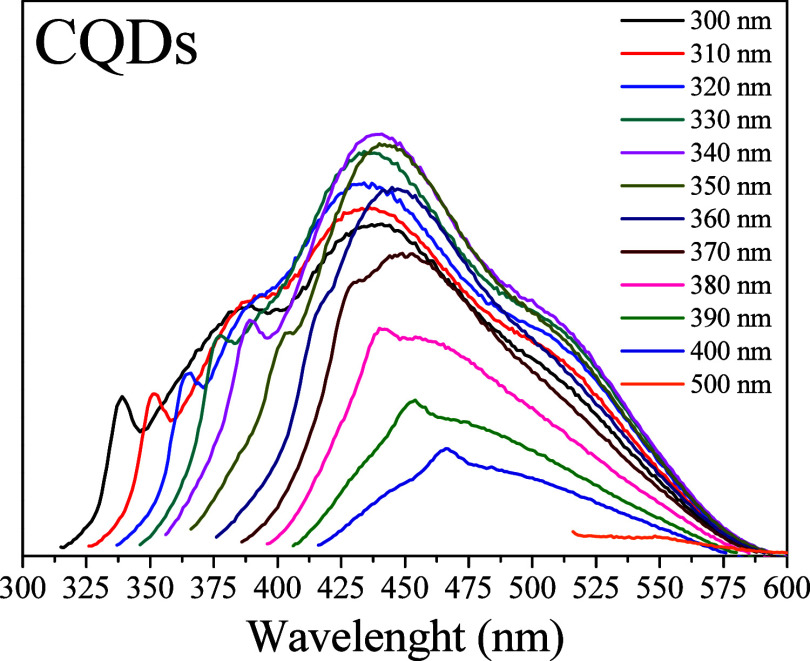
CQD emission spectrum by UV light excitation between 300 and 500 nm.

The elemental composition is a crucial aspect for the application of CQDs as corrosion inhibitors, as the presence of functional groups can facilitate the adsorption of CQDs on the metal surface, thereby directly influencing the inhibitory activity.^7,8,28^ The chemical and structural information on CQDs synthesized from garlic peel can be observed in the FTIR spectrum (Figure 5). From the spectrum, it is possible to perceive several bands in the garlic peel and the CQD synthesized from it. A broad and intense band is observed in both spectra at 3440 cm^–1^, indicating the presence of axial deformation vibrations of O–H and N–H, which suggests the existence of amino and/or hydroxyl groups on the CQD surface.^35–37^ The band at 2930 cm^–1^ can be associated with the axial deformation vibration of the C–H bond.^35–38^ The band wavelengths of 1620 and 1425 cm^–1^ correspond to the vibrations of C=C bonds, indicating the presence of aromatic ring systems within the CQD structure.^26^ The bands at 1425 and 1620 cm^–1^ can also be associated with the vibrations of the axial deformation of the C=N bond and the carbonyl group (C=O), respectively.^26,38,39^ Additionally, a small band at 1325 cm^–1^ is likely related to the axial vibration of the N–H bond.^40^ The intense band seen in the CQD spectrum at 1130 cm^–1^ is associated with the vibration of the C–O bond.^40^ The vibration associated with the axial distortion of the SO–O bond corresponds to a wavenumber of 1115 cm^–1^.^41^ Additionally, the band observed around 620 cm^–1^ can be linked to the angular distortion vibration of the =C–H bond.^38^ The FTIR analysis provide evidence for the presence of hydrophilic groups, which contribute to the solubility of the synthesized carbon quantum dots (CQDs) in water, which is a desirable characteristic of the material. Furthermore, this analysis indicates that the chemical composition of the raw materials was effectively transferred to the synthesized CQDs. This key aspect will be examined in more detail through XPS measurements.

**Figure 5:**
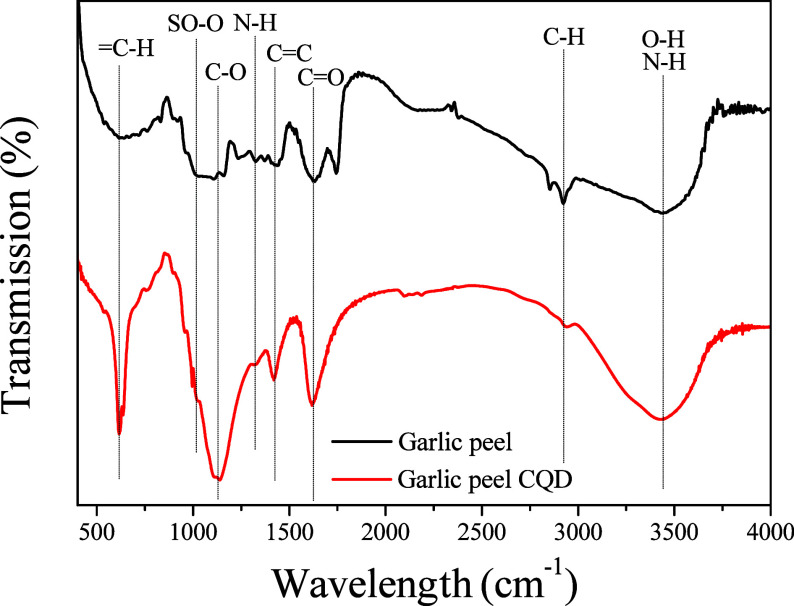
FTIR spectra of garlic peel and synthesized CQD.

Figure 6A displays the survey spectrum, which reveals the presence of carbon (C), nitrogen (N), oxygen (O), and sulfur (S) in the chemical compositions of carbon quantum dots (CQDs). To ascertain whether these elements are integral to the material structure, high-resolution measurements of C 1s, N 1s, O 1s, and S 2p were conducted (Figure 6B,C,D,E, respectively). These high-resolution measurements, particularly for C 1s and N 1s, demonstrate chemical interactions between C–S, C–N, and C=O. This indicates that during the synthesis of CQDs, the chemical elements present in the biomass were incorporated into the CQD structure through a process known as endogenous doping. Nitrogen, sulfur, and oxygen are components of organic compounds found in garlic skin. For instance, alliin and allicin serve as a source of nitrogen and sulfur; carbohydrates are primary sources of carbon and oxygen. Additionally, it is essential to note that the sulfur detected in the CQDs may have also been introduced by H_2_SO_4_ during the acid hydrolysis of garlic peel at 200 °C. Table 1 summarizes the binding energies associated with the interactions depicted in Figure 6.

**Figure 6:**
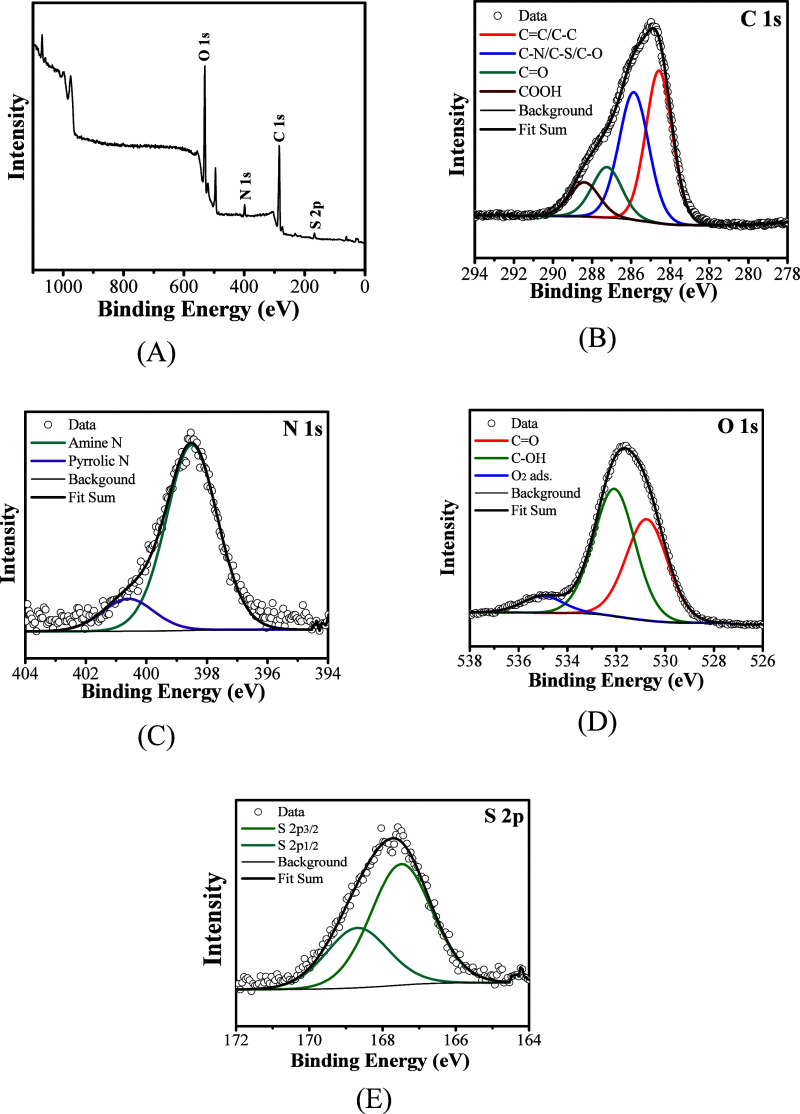
X-ray photoelectron spectroscopy (XPS) spectroscopy survey of the synthesized CQDs (A) and high-resolution measurements of C 1s (B), N 1s (C), O 1s (D), and S 2p (E).

**Table 1:** Data Obtained from High-Resolution XPS Measurements of C 1s, N 1s, O 1s, and S 2p Found in the Synthesized CQDs

element	group	binding energy (eV)	ref
**C 1s**	C=C/C–C	284.6	^42^
	C–N/C-S/C–O	285.8	^43^
	C=O	287.3	^44^
	COOH	288.4	^45^
**N 1s**	amine N	398.5	^44^
	pyrrolic N	400.6	^44^
**O 1s**	C=O	530.8	^43^
	C–OH	532.1	^42^
	OH	535	^43^
**S 2**	S 2p3/2	167.5	^42^
	S 2p1/2	168.7	^42^

Characterization results confirm the successful synthesis of CQDs from garlic peel via acid hydrolysis. This process highlights the incorporation of functional groups and heteroatoms from garlic peel, making these CQDs promising corrosion inhibitors.

### 4.2 Corrosion Analysis

#### 4.2.1 Gravimetric Tests

Table 2 presents the results of the 2 and 24 h immersion tests in the absence and presence of corrosion inhibitor at concentrations of 50, 100, 200, 300, and 400 ppm in 1 mol L^–1^ HCl 1 mol L^–1^. For both times evaluated, a successive decrease in the corrosion rate is observed as the inhibitor concentration is increased, indicating the formation of a film that protects the mild steel against the corrosive environment. For equal inhibitor concentrations at times of 2 and 24 h, it is observed that their corrosion rates decrease drastically, indicating that the formation of a protective layer is favored with a time of at least 24 h. Similar inhibition efficiencies were observed for 300 and 400 ppm concentrations, with 79% inhibition after 2 h. For the 24 h, identical inhibition efficiencies are observed for the 200, 300, and 400 ppm concentrations, with 96% inhibition, indicating saturation of the inhibitor at 200 ppm.

**Table 2:** Gravimetric Measurements for the Mild Steel in 1 mol L^–1^ HCl in the Presence and Absence of CQDs at 2 and 24 h at Room Temperature

		2 h		24 h	
medium	concentration (ppm)	*W*_corr_ (mg cm^**–**2^ h^**–**1^)	IE (%)	*W*_corr_ (mg cm^**–**2^ h^**–**1^)	IE (%)
**blank**		1.62		1.80	
**CQDs**	50	0.849	47.6 ± 0.99	0.492	72.7 ± 1.32
	100	0.708	56.3 ± 0.40	0.0952	94.7 ± 0.05
	200	0.466	71.2 ± 1.91	0.0748	95.8 ± 0.37
	300	0.341	79.0 ± 0.36	0.0698	96.1 ± 0.30
	400	0.337	79.2 ± 0.62	0.0779	95.7 ± 0.29

Table 3 compares the maximum corrosion inhibition efficiency of the CQD produced from garlic peel with that of CQDs from other carbon sources. In comparative terms, the synthesized CQD obtained a good maximum inhibition efficiency, presenting a result similar to the other CQDs.

**Table 3:** Comparison of Garlic Peel CQD Maximum Corrosion Inhibition Efficiency with Other Carbon Quantum Dots Studied in the Literature for Mild Steel in 1 mol L^–1^ HCl

material	optimum concentration (ppm)	maximum efficiency (%)	ref
pitaya peel-derived N,S-CQDs	200	95.6	^14^
grapefruit peel-derived CQDs	200	94.1	^15^
folic acid and o-phenylenediamine-derived N-CQDs	150	95.4	^46^
citric acid and dodecylamine-derived N-CQDs	150	92.2	^47^
lupine-derived N-CQDs	175	89.3	^48^
sugar cane bagasse CQDs	100	94.0	^49^
pumpkin seeds CQDs	10	94.6	^50^
garlic peel CQDs	200	95.8 ± 0.37	This work

Gravimetric tests with temperature variation were conducted to assess the impact of the temperature on inhibition. Table 4 presents the results obtained for gravimetric tests in the absence and presence of an inhibitor at a fixed concentration of 200 ppm for temperatures of 25, 35, 45, and 55 °C.

**Table 4:** Gravimetric Measurements for Mild Steel in 1 mol L^–1^ HCl in the Absence and Presence of 200 ppm of CQDs for 2 h at 25, 35, 45, and 55 °C Temperatures

	temperature							
	25 °C		35 °C		45 °C		55 °C	
medium	*W*_corr_ (mg cm^**–**2^ h^**–**1^)	IE (%)	*W*_corr_ (mg cm^**–**2^ h^**–**1^)	IE (%)	*W*_corr_ (mg cm^**–**2^ h^**–**1^)	IE (%)	*W*_corr_ (mg cm^**–**2^ h^**–**1^)	IE (%)
**Blank**	1.62		3.03		5.90		8.57	
**CQDs**	0.466	71.2 ± 1.61	0.682	77.5 ± 0.63	2.24	62.0 ± 1.48	4.57	46.7 ± 1.27

As shown, corrosion rates increase with increasing temperature for both media with and without inhibitors, with a decrease in corrosion rate in the presence of inhibitors at all temperatures. An increase in the inhibition efficiency is also observed between temperatures of 25 and 35 °C and a notable decrease in the inhibition efficiency from 45 °C onward. The observed reduction can be attributed to the increase in the desorption rate as the temperature rises.^51^ The interactions between the quantum dot and the surface can be chemical, physical, or mixed, with the physical being the weakest.^52^ To study which interaction occurs in the adsorption, the Arrhenius equation was used (eq 6),^52^ where the activation energy can be obtained by the linear regression between the logarithm of corrosion rate and the inverse of the temperature (Figure 7) using the data in Table 4:

$$\ln\;W=\ln\;A-\frac{\mathrm{Ea}}{RT}$$ 6

**Figure 7:**
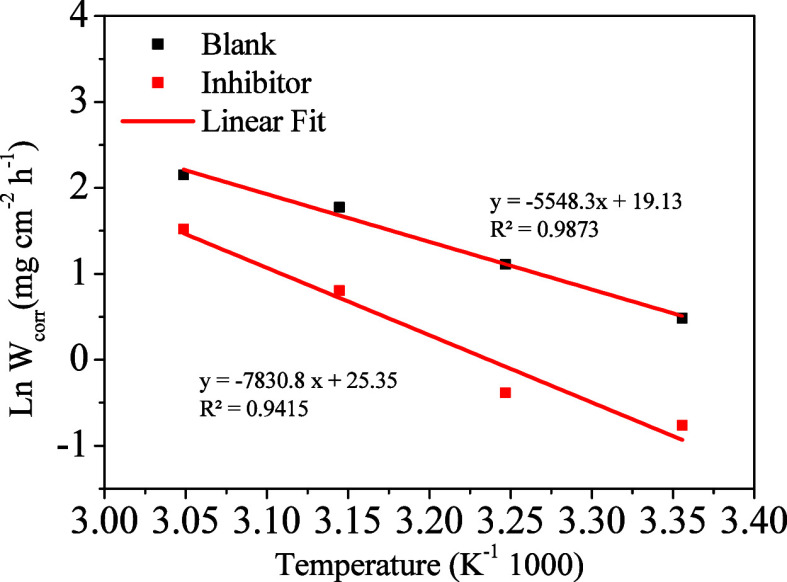
Arrhenius plot for mild steel in 1 mol L^–1^ HCl in the absence and presence of 200 ppm of CQDs at temperatures 25, 35, 45, and 55 °C during 2 h of immersion.

*W* is the corrosion rate, *A* is the Arrhenius constant, Ea is the activation energy, *R* is the gas constant, and *T* is the temperature.

The thermodynamic activation parameters, such as activation energy (Ea), help us to understand the kinetics and mechanism of corrosion. The activation energy obtained in the presence of an inhibitor was 65.1 ± 0.53 kJ/mol, while in its absence, it was 46.1 ± 0.22 kJ/mol. These results suggest that CQD interacts with the surface of mild steel through electrostatic interactions,^53^ corroborating the behavior observed in Table 4.

#### 4.2.2 Electrochemical Tests

To perform electrochemical tests and study the influence of the inhibitor, the system must be in a steady state. The open-circuit potential (OCP) graph versus immersion time (Figure 8) shows that up to 7200 s, all systems are in a steady-state OCP. Furthermore, it is observed that in the presence of the inhibitor, as the concentration increases, there is a shift of the OCP to more anodic potentials, indicating that the inhibitor influences the electrochemical processes at the MS surface.^54^

**Figure 8:**
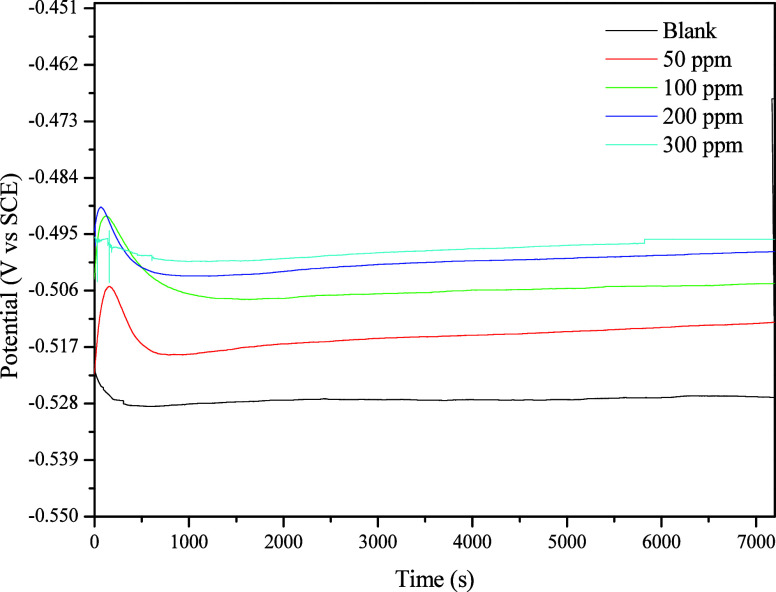
Open-circuit potential graphs for mild steel in 1 mol L^–1^ HCl in the absence and presence of different concentrations of CQDs.

The Nyquist and Bode plots obtained by electrochemical tests are shown in Figure 9. It is possible to observe a capacitive loop in the medium without an inhibitor (Figure 9A), which is directly linked to the capacitance of the electric double layer and the charge transfer resistance.^55^ With the addition and increase in inhibitor concentration, the loop tends to grow, indicating the formation of a protective film of the inhibitor on the mild steel surface capable of protecting it against the action of H^+^ and Cl^–^ ions. An inductive loop can also appear with the addition of the inhibitor, primarily at high inhibitor concentrations (300 ppm), which is associated with the relaxation of species, such as (FeH)_ads_ and FeOH_ads_. Both species are intermediates of cathodic and anodic reactions and can interact with the inhibitor, influencing the corrosion inhibition efficiency.^56,57^ Another possibility for an inductive loop in systems with inhibitors is relaxation of the inhibitory molecule onto the metallic surface.

**Figure 9:**
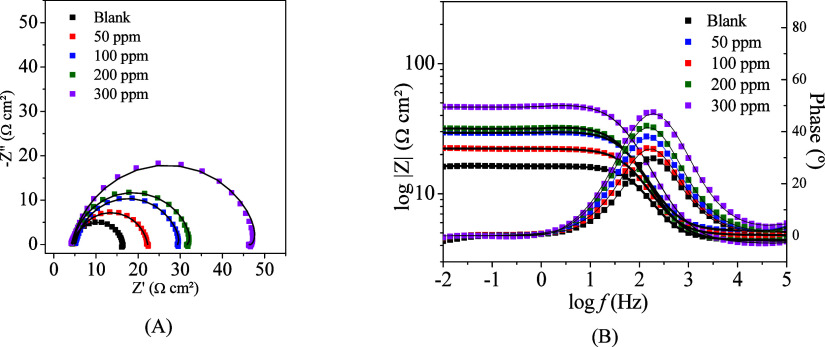
Nyquist diagrams (A) and Bode plots (B) of mild steel in 1 mol L^–1^ HCl in the absence and presence of CQDs at 50, 100, 200, and 300 ppm.

At low frequencies, the impedance modulus increases with increasing inhibitor concentration (Figure 9B), indicating that at higher concentrations, a more significant amount of CQDs is adsorbed on the surface of the mild steel, which hinders charge transfer between the MS and the solution. As with the impedance modulus, the phase angle also increases with increasing inhibitor concentration, reaching a value close to 50° at 300 ppm, once again suggesting the formation of a protective layer due to the more capacitive behavior.^58^

Table 5 summarizes the electrochemical parameters obtained by the equivalent circuit of the Nyquist diagram presented in Figure 10.

**Figure 10:**
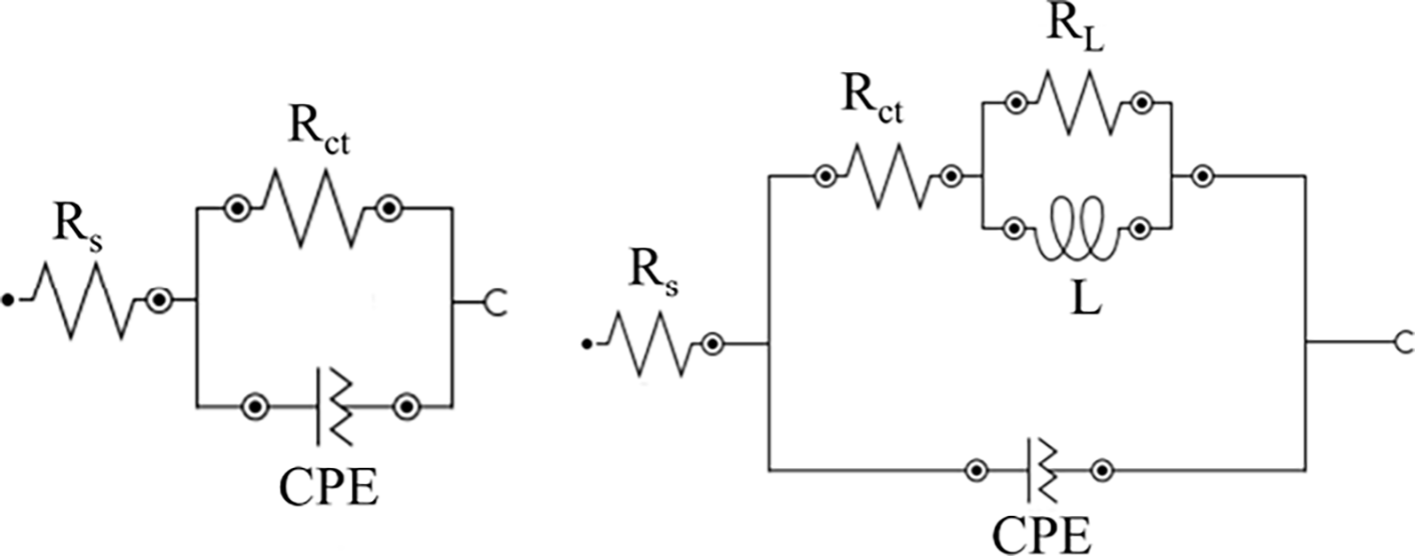
Electrochemical circuit used to extract the electrochemical parameters from the Nyquist diagram.

**Table 5:** Electrochemical Data Obtained by Adjusting the Impedance Using the Equivalent Circuit Presented in Figure 10

medium	concentration	***R**_**s**_* **(Ω cm** **^2^** **)**	*C*_dl_ (μF cm^–2^)	*R*_ct_ **(Ω cm** **^2^** **)**	***R**_**p**_* **(Ω cm** **^2^** **)**	*L* (H cm^2^)	*R*_*L*_ **(Ω cm** **^2^** **)**	*X*^2^ × 10^*3*^	IE (%)
**blank**		4.42	154	11.91	11.91			0.12	
**CQD**	50	4.53	130	17.50	17.50			0.13	31.9
	100	5.15	108	24.88	24.45	0.1	0.63	0.29	52.1
	200	4.42	111	28.48	27.35	0.182	1.27	0.34	58.2
	300	4.21	74.6	41.70	37.92	0.4	2.96	0.68	71.4

Where *R_s_* and *R*_ct_ are the solution resistance and charge transfer resistance, respectively; *L* and *R_L_* are the components of the inductive elements, and due to the heterogeneity of the surface, the constant phase element (CPE) was used instead of a pure capacitor to model the electrical double layer (EDL) at the interface between the MS electrode and the electrolyte.

As the inhibitor concentration increases, the double-layer capacitance (*C*_dl_) decreases, and the charge transfer resistance (*R*_ct_) increases. *C*_dl_ represents the amount of charge stored in the metal-solution interface, which directly affects the ability of charge transfer between the material and the solution.^59,60^ The decrease in capacitance indicates the formation of a protective layer that prevents the attack of H^+^ ions against the metal surface.^61,62^ Table 5 also presents the inhibition efficiency (IE) results in percentage. The IE values from *R*_ct_ tended to increase, corroborating the mass loss results with the increase in the CQD concentration. The inhibitor adsorption kinetics, as seen in gravimetric assays for 2 and 24 h, is slow, making time a crucial factor in the adsorption of the produced material. The impedance diagrams were obtained after a 2 h immersion time.

The electrical double layer (*C*_dl_) was obtained from eq 7:^63^

$$C_{\mathrm{dl}}=Y_{0}(2\pi f_{\max}{)}^{n-1}$$ 7

*Y*_0_ is the magnitude of CPE, *n* represents the deviation from the ideal behavior falling between −1 and 1, and *f*_max_ is the frequency at which the imaginary component of the impedance is maximal.

Polarization curves are essential for understanding the corrosion mechanism, as the inhibitor may inhibit corrosion by blocking anodic and/or cathodic reactions.^6^ Figure 11 shows the effect of the addition of CQD on the potentiodynamic polarization of mild steel in an acidic medium. As shown, the presence of CQDs results in a notable decrease in current density in both the cathodic and anodic regions, thus characterizing it as a mixed-type inhibitor with a predominance of inhibition in the anodic region.^6,64–66^ These results corroborate the shift in the OCP and the corrosion potential to a more positive potential.

**Figure 11:**
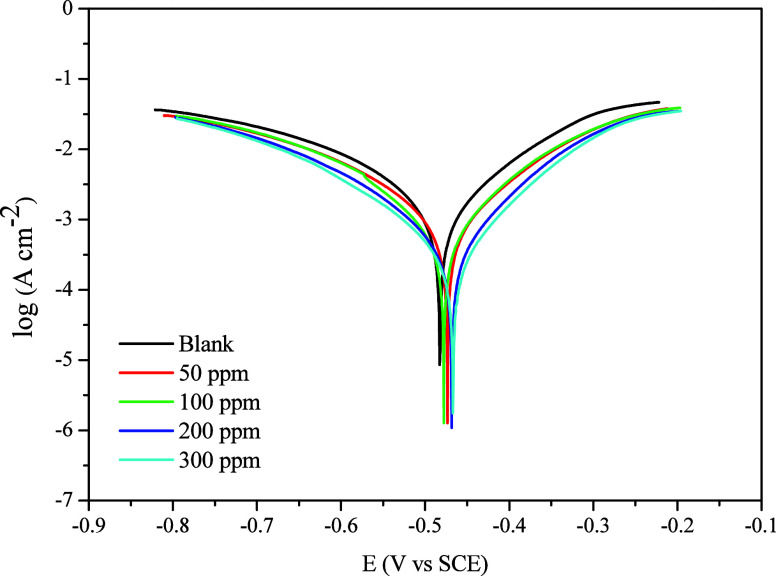
Electrochemical polarization curves for mild steel in 1 mol L^–1^ HCl in the presence and absence of the inhibitor.

The Tafel extrapolation of the polarization curves provided the key electrochemical parameters summarized in Table 6. A gradual shift in the corrosion potential (*E*_corr_) toward more positive values was observed with increasing CQD concentration, indicating the predominant anodic inhibition activity. Moreover, as the CQD concentration increased up to 300 ppm, an apparent decrease in *J*_corr_ was observed, from 1.022 to 0.327 mA cm^–2^, corresponding to an increase in inhibition efficiency up to 68.0%, highlighting the effectiveness of CQDs in suppressing the corrosion process. This trend is consistent with the *R*_*p*_ values, which increased from 22.9 to 81.3 Ω cm^2^ after adding 300 ppm of CQD, also showing a rising IE by *R_p_* of up to 71.8%, which closely matches the impedance-based efficiency of 71.4% from Table 5.

**Table 6:** Results Obtained from the Tafel Extrapolation of the Polarization Curve for Mild Steel in 1 mol L^–1^ HCl in the Absence and Presence of CQDs at Concentrations of 50, 100, 200, and 300 ppm

[CQD] (ppm)	OCP (mV/ECS)	*E*_corr_(mV/ECS)	*J*_corr_(mA cm^–2^)	IE by *J*_corr_ (%)	β_*c*_ (V/dec)	β_*a*_ (V/dec)	*R*_*p*_ **(Ω cm** **^2^** **)**	IE by *R_p_* (%)
blank	–527	–483	1.022		–0.087	0.099	22.9	
50	–512	–473	0.688	32.7	–0.088	0.106	30.2	24.0
100	–505	–479	0.598	41.5	–0.102	0.141	46.3	50.4
200	–498	–469	0.406	60.3	–0.104	0.171	55.9	58.9
300	–496	–467	0.327	68.0	–0.113	0.189	81.3	71.8

The Tafel slopes (β_a_ and β_c_) revealed that CQDs affected both anodic and cathodic reactions, confirming the mixed-type inhibition behavior region.^6,64–66^ At 300 ppm, β_a_ increased to 0.189 V/dec, while β_c_ decreased to −0.113 V/dec. These results show that the anodic Tafel slope is more modified than the cathodic Tafel slope with inhibitor addition.

In summary, both EIS and Tafel polarization studies demonstrate that CQDs derived from garlic peel effectively adsorb onto the mild steel surface, hindering electron transfer processes and significantly lowering corrosion rates. The convergence of inhibition efficiencies calculated by *R*_*p*_, *R*_ct_, and *J*_corr_ validates the consistency and reliability of the CQDs’ corrosion inhibition performance.

The results obtained from gravimetric and electrochemical analyses demonstrate a strong agreement, collectively confirming the effective corrosion mitigation imparted by CQDs on mild steel surfaces. The consistent trends in mass loss reduction and electrochemical parameters highlight the inhibitor’s robust protective performance in acidic environments.

### 4.3 Adsorption Isotherms

Adsorption isotherms are valuable models for obtaining information about the adsorption mechanism. Among them are the number of adsorbed inhibitor molecules, the number of layers formed, and the adsorption equilibrium constant of the inhibitor.^52^ The isotherms used in this journal were Langmuir, Temkin, Flory–Huggins, and El-Awady, represented by eqs 8, 9, 10, and 11, respectively:

$$\log\left(\frac{\theta }{1-\theta }\right)=\log K+\log C$$ 8

$$\theta =\left(\frac{-2.303}{2a}\right)\log K+\left(\frac{-2.303}{2a}\right)\log C$$ 9

$$\log\left(\frac{\theta }{C}\right)=\log K+x\log\left(1-\theta \right)$$ 10

$$\log\left(\frac{\theta }{1-\theta }\right)=\log K+y\log C$$ 11

where *C* is the inhibitor concentration (mg L^–1^), *K* is the adsorption equilibrium constant, *a* is the lateral interaction parameter between the adsorbed molecules, *x* is the number of adsorbed water molecules replaced by inhibitor molecules, *y* is the number of inhibitor molecules adsorbed at each active site, and θ is the degree of surface coverage of the inhibitor molecule calculated by $\frac{\mathrm{EI}}{100}$ .

Table 7 presents the equations of the four isotherms by the linear regression of the data obtained by the gravimetric tests.

**Table 7:** Langmuir, Temkin, Flory-Huggins, and El-Awady Isotherm Equations Obtained by Linear Regression of Gravimetric Test Results for a 2 h Immersion Time

isotherm	equation	*R* ^2^
**Langmuir**	*y* = 1.1213*x* + 53.19	0.9964
**Temkin**	*y* = 0.3544*x* – 0.1136	0.9664
**Florry-Huggins**	*y* = 1.5356*x* – 1.6091	0.9305
**El-Alwady**	*y* = 0.7064*x* – 1.2328	0.9621

Among the isotherms evaluated, only the Langmuir isotherm presents *R* ^2^ above 0.99. The Langmuir isotherm assumes the formation of a monolayer of inhibitor molecules on the metal surface, without lateral interactions between adsorbed molecules, which suggests the preferential interaction between CQD and the MS surface, a desirable characteristic for a corrosion inhibitor.^52,67^ However, the other isotherms presented *R* ^2^ values above 0.9, which cannot be ignored. Moreover, the slope of the Langmuir equation must be 1, and the experimental value is 1.1213, which shows a deviation from the Langmuir model. The fact that all models studied yield *R* ^2^ values above 0.9 is linked to the complex chemical composition and variations in the sizes of the synthesized CQDs, which enable different types of interaction.

### 4.4 Surface Characterization

Characterization techniques are essential tools for corroborating the results obtained in corrosion tests and observing the influence of the inhibitor’s presence on the metal surface. While scanning electron microscopy and atomic force techniques provide information on the surface morphology and roughness, angle measurements provide information on the surface hydrophobicity and the influence of the film formed. Figure 12 shows the different contact angles of water droplets on the surface of a freshly polished mild steel (Figure 12A) and after immersion tests in 1 mol L^–1^ HCl in the absence (Figure 12B) and presence (Figure 12C) of the inhibitor. After immersion in the acidic medium without the inhibitor, a decrease in the contact angle of the water droplets from 69° to 38° can be observed (Figures 12A,B), indicating an increase in the hydrophilic character of the surface.^68,69^ This effect is explained by the increase in surface roughness after corrosion in an acidic medium, which generates a larger surface area (Figures 12A,B) and by the formation of inorganic corrosion products that alter the surface properties and can increase surface wettability.^40,68^ In contrast, the water droplet in contact with the mild steel after immersion in an acidic medium with an inhibitor increased the angle to 80° (Figure 12C). In this case, the increase in the contact angle indicates an increase in surface hydrophobicity, which can be explained by the formation of a hydrophobic film composed of CQDs.

**Figure 12:**
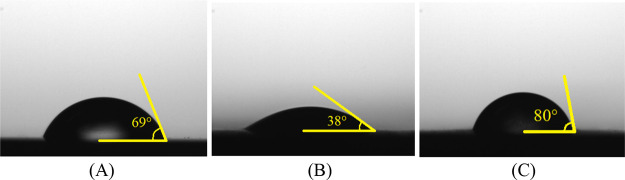
Contact angle of a water drop on MS: freshly polished (A), after 2 h of immersion in 1 mol L^–1^ HCl in the absence of inhibitor (B) and after 2 h of immersion in 1 mol L^–1^ HCl in the presence of the inhibitor (C).

The surface of MS before and after immersion in an acidic medium is best observed by SEM (Figure 13). The surface of the freshly polished steel exhibits a smooth texture with parallel scratches characteristic of MS surface preparation (Figure 13A). After immersion in HCl 1 mol L^–1^ without an inhibitor, the mild steel surface becomes rough without the scratches from the abrading process (Figure 13B), caused mainly by the dissolution of iron.^70^ However, in the presence of inhibitors, the surface presented less roughness after immersion in an acidic medium (Figure 13C), and parallel scratches from abrasion are still visible. Thus, it is possible to observe the ability of the synthesized CQDs to inhibit the corrosion of mild steel, therefore preventing iron attack by the acidic medium, which causes an increase in roughness.

**Figure 13:**
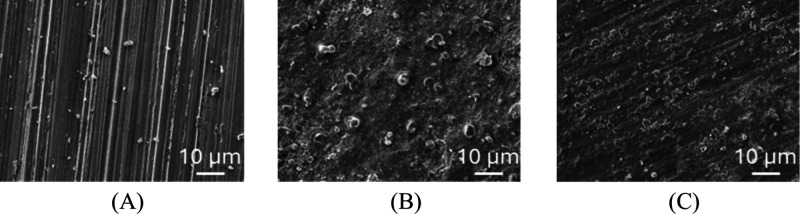
SEM images of mild steel: freshly polished (A), after 2 h of immersion in 1 mol L^–1^ HCl in the absence of the inhibitor (B) and after 2 h of immersion in 1 mol L^–1^ HCl in the presence of the inhibitor (C).

The rough surface of the mild steel was calculated by using AFM measurements (Figure 14). As shown, the freshly polished MS (Figure 14A) presented an RMS value of ±27 nm, while for the MS after immersion in 1 mol L^–1^ HCl in the absence of the inhibitor (Figure 13B) and presence (Figure 14C), RMS values of 273 and 243 nm were obtained, respectively. As already mentioned, the increase in roughness is directly linked to the dissolution of iron caused by the corrosive medium. Therefore, these values support the SEM measurements, indicating that the metal surface is better preserved in CQDs, which corroborates the results of the gravimetric and electrochemical corrosion studies.

**Figure 14:**
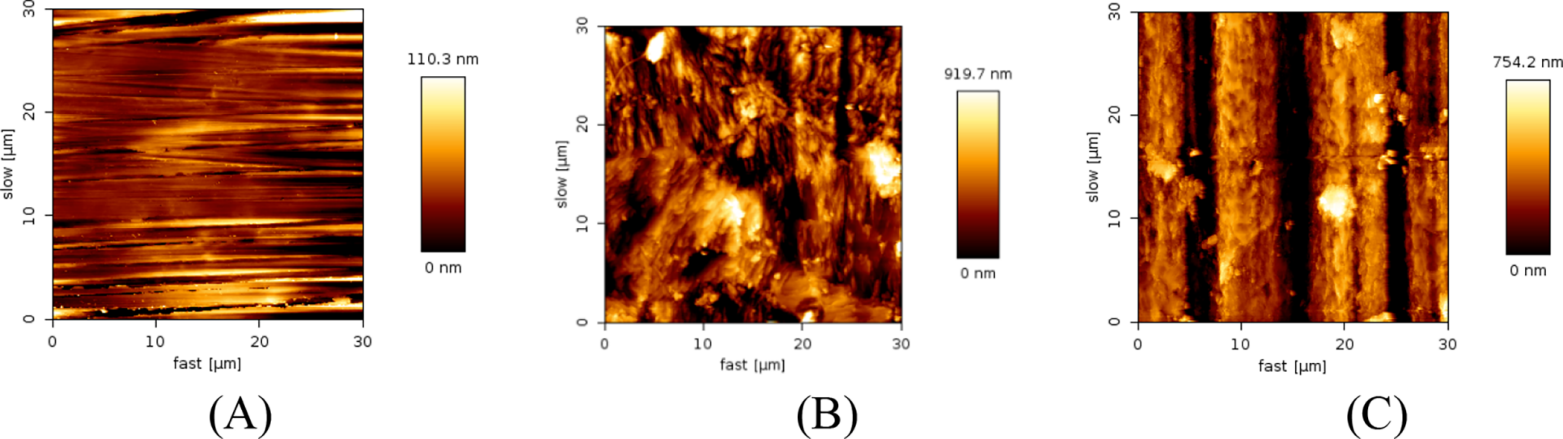
AFM images of mild steel: freshly polished (A), after 2 h of immersion in 1 mol L^–1^ HCl in the absence of the inhibitor (B) and after 2 h of immersion in 1 mol L^–1^ HCl in the presence of the inhibitor (C).

To study the inhibitor film formation and its influence on the metal surface, X-ray photoelectron spectroscopy was performed on the mild steel plate after immersion testing in hydrochloric acid without the inhibitor and in the presence of a 400 ppm inhibitor (Figure 15). As can be observed, in the survey spectrum (Figure 15A) both in the absence and presence of the inhibitor, the elements Fe, O, and C were found, thus achieving high resolution of the species Fe 2p (Figure 15B,C), C 1s (Figure 15D,E), and O 1s (Figure 15F,G).

**Figure 15:**
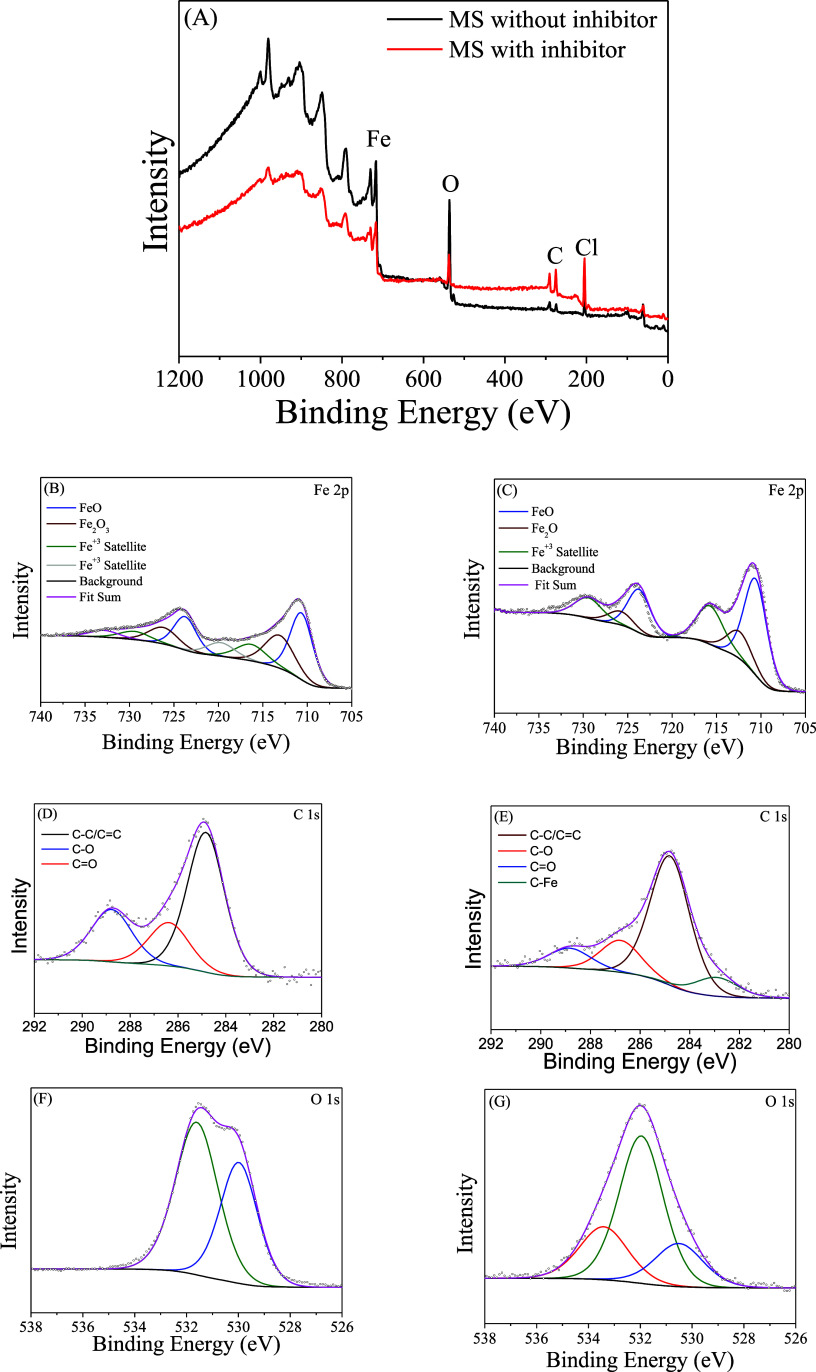
Survey spectrum of mild steel after immersion corrosion tests in the presence (A) and absence (B) of carbon quantum dots and high-resolution spectrum of Fe 2p, C 1s, and O 1s in the absence of CQDs (B,D,F, respectively) and presence of carbo quantum dots (C,E,G, respectively).

Table 8 presents the binding energies observed in the spectrum and the chemical composition of the mild steel surface in the absence and presence of the inhibitor. Observing the chemical composition, the influence of the inhibitor is already evident. As shown in Figure 6, the inhibitor used is rich in carbon and oxygen, with traces of nitrogen and sulfur. In the sample with the inhibitor, we did not observe the presence of nitrogen and sulfur, which is understandable since the quantity of nitrogen and sulfur in the sample is very small. However, it is observed that when compared to the sample without inhibitor, the carbon concentration increases significantly while the oxygen concentration decreases. This phenomenon demonstrates the presence of the adsorbed inhibitor and its inhibitory effect.

**Table 8:** Data Obtained from Survey Spectra and High-Resolution XPS Measurements of Fe 2p, C 1s, and O 1s Found in the Surface of Mild Steel after Immersion in HCl in the Presence and Absence of Synthesized CQD

sample	element	binding energy	bonding	ref
**mild steel without CQD chemical composition: C: 13.7% O: 57.3% Fe: 29%**	Fe 2p_3/2_	710.64	FeO	^72^
		713.10	Fe_2_O_3_	^72^
		716.33	Fe^3+^ satellite	^72^
		719.74	Fe^3+^ satellite	^71^
	C 1s	284.83	C–C/C=	^72^
		286.38	C–O	^76^
		288.77	C = O	^72^
	O 1s	529.98	FeO/Fe_2_O_3_	^77^
		531.61	FeOOH	^78^
**mild steel with CQDs chemical composition: C: 48.6% O: 32.2% Fe: 19.2%**	Fe 2p_3/2_	710.59	FeO	^72^
		712.42	Fe_2_O_3_	^72^
		715.73	Fe^3+^ satellite	^72^
	C 1s	282.91	C–Fe	^75^
		284.80	C–C/C=C	^78^
		286.78	C–O	^76^
		288.83	C=O	^72^
	O 1s	530.51	FeO/Fe_2_O_3_	^77^
		531.96	FeOOH	^72^
		533.41	OH	^79^

The significant increase in carbon in the sample with the inhibitor compared with the sample without the inhibitor indicates that the inhibitor is adsorbed on the metal surface. Additionally, the inhibitor is rich in oxygen, and despite this, the oxygen content decreases; this indicates that during the immersion process, the formation of iron oxides was mitigated, which demonstrates the inhibitory effect of the carbon quantum dot. This becomes more evident when we analyze the high-resolution peaks of Fe 2p_3/2_. When we compare the systems, we observe the following behavior. When the inhibitor is added to the system, the peak corresponding to Fe^3+^ Satellite at 719.74 eV^71^ disappears while the peak at 716.33 eV^72^ increases and the peak at 713.10 eV^72^ decreases significantly. This result suggests that there is an interaction between Fe^3+^ and the inhibitor.

Furthermore, a shift in binding energies is observed, where the peak for Fe^3+^ satellite shifts from 716.33 to 715.73 eV and from 713.10 to 712.41 eV for Fe_2_O_3_. The spectrum without inhibitor, at 719 eV, which corresponds to Fe^3+^, indicates that iron is highly oxidized.^73^ When it disappears in the presence of the inhibitor, it suggests that the carbon quantum dot is inhibiting iron corrosion.^74^ Additionally, the increase in the 715.73° peak, which may also be related to Fe^3+^, is associated with the decrease in the peak for Fe_2_O_3_. This suggests that some interaction with Fe^3+^ is occurring, intensifying its binding energy, even in smaller quantities. Furthermore, the shift in binding energy of approximately 0.6 eV for both may indicate that the surface is being protected from the oxidation process of Fe^2+^ to Fe^3+^ or that a bond has formed between iron and the inhibitor elements, which affects the electron density of Fe. For this, it is essential to evaluate the high-resolution carbon. The groups in the high-resolution C do not differ significantly whether or not an inhibitor is present. The groups are the same, but the amount of carbon in the presence of the inhibitor is more significant, which already indicates the presence of CQD on the surface. However, an element binding energy appears in the presence of the inhibitor at very low intensity, which is designated as a bond between iron and carbon (C–Fe).^75^ This bond is valid for the discussion regarding Fe spectra and confirms the presence of the inhibitor through a chemical interaction with iron.

The XPS study corroborates surface morphological characterization studies and corrosion tests, showing that the carbon quantum dot adsorbed on the mild steel surface could mitigate corrosion. Additionally, we observed a chemical interaction between the inhibitor and the metal surface, even at low intensity, which contradicts the results of the Arrhenius study. This shows the importance of meticulous interpretation of the results and the significance of the XPS technique. This contradiction is unsurprising since the carbon quantum dot is a structure with a complex chemical composition. We must remember that XPS is very sensitive to chemical interactions. These results indicate that the inhibitor exhibits two types of interactions, predominantly physical, and a small fraction involves chemical interactions, which is entirely plausible given the chemical composition of the quantum dots.

### 4.5 Adsorption Mechanism

The understanding of the adsorption mechanism of carbon quantum dots derived from garlic peel onto the surface of mild steel in an acidic medium was deepened through dynamic light scattering and zeta potential analyses. According to DLS results in DMF, the synthesized CQDs present sizes below 10 nm, which would reduce the energy barrier and increase surface contact due to their small dimensions.^80,81^ Consequently, the material tends to agglomerate, leading to a decrease in the system’s free energy.^80,81^ The chemical composition and diversity of functional groups present in the material also favor agglomeration,^82^ as observed in the CQD characterization results.

The DLS results in acidic medium are shown in Figure 16A,B. At the initial time (*t*_0_) and 50 ppm concentration, two distinct peaks were observed, with average hydrodynamic diameters of 1.13 and 393.5 nm (Figure 16A). After 2 h of immersion in 1 mol L^–1^ HCl (*t*_2_), a different distribution appeared, with two prominent peaks at 296.6 and 1731.2 nm (Figure 16A). The 1.13 nm peak likely corresponds to the freeCQDs at *t*_0_, while the 393.5 nm peak indicates partial agglomeration of suspended CQDs. After 2 h, the small-size peak disappears, and two peaks greater than 100 nm are observed, indicating complete agglomeration of the material. Additionally, a trend toward more extensive agglomerate formation over time is evident from the appearance of the 1731.2 nm peak at *t*_2_.

**Figure 16:**
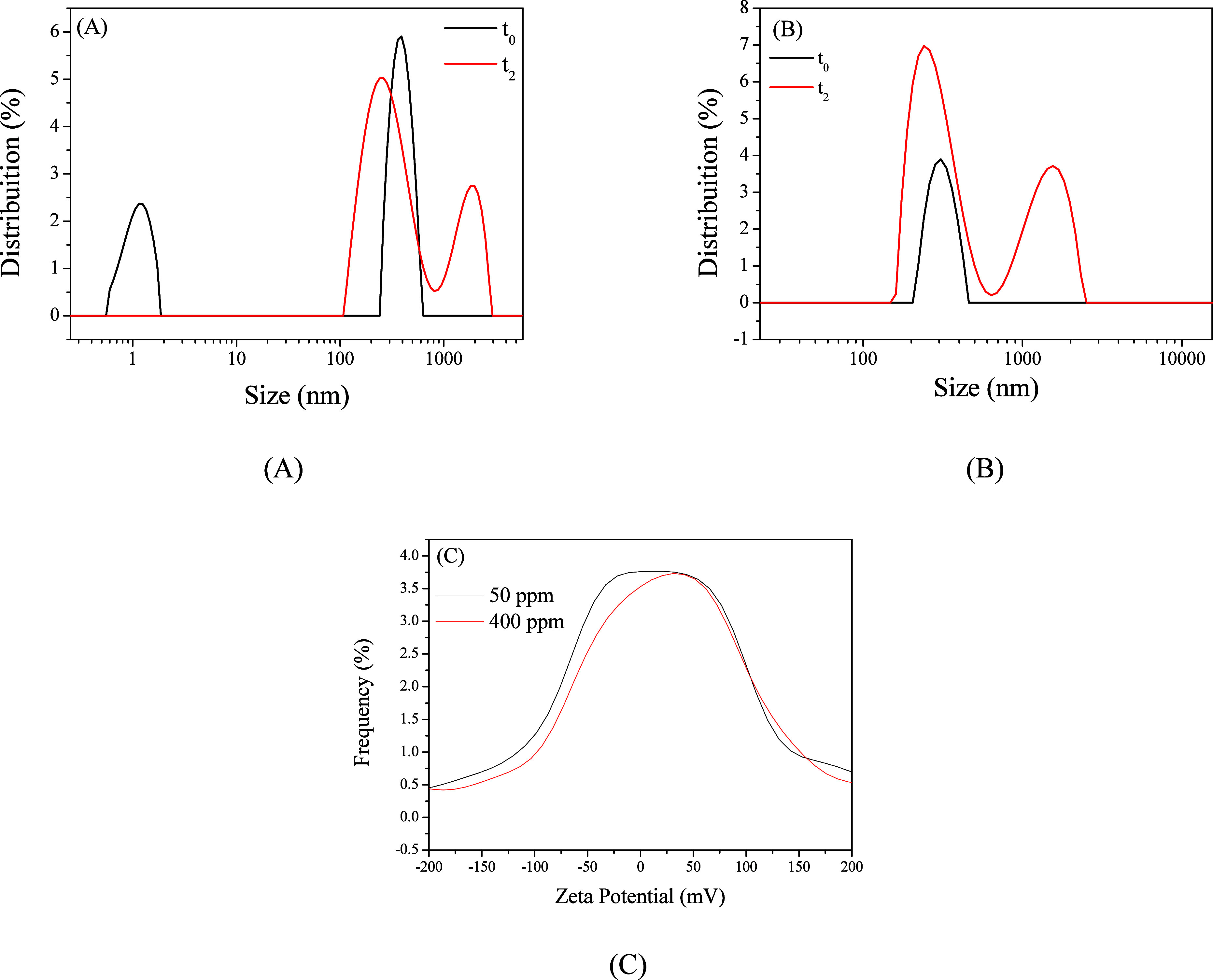
DLS analysis of produced CQDs in 1 mol L^–1^ HCl at *t*_0_ and after 2 h at concentrations of 50 ppm (A) and 400 ppm (B) and zeta potential of CQDs at concentrations of 50 and 400 ppm at *t*_0_ (C).

For the 400 ppm concentration, a different behavior was observed. At *t*_0_, a peak with an average hydrodynamic diameter of 311.5 nm was identified (Figure 16B). After 2 h, two peaks at 283.5 and 1454.4 nm were observed (Figure 16B). Unlike the 50 ppm behavior at *t*_0_, no free CQDs were detected at 400 ppm, as there were no peaks below 10 nm. This suggests that CQDs tend to agglomerate more readily at higher concentrations. Like the 50 ppm condition, a larger-sized particle peak appears after 2 h, again indicating a tendency toward more significant agglomerate formation over time. These findings suggest that even after 2 h, CQDs at high concentrations maintain a distribution of relatively large agglomerates, implying that these aggregates are sufficiently stable to form a thick and continuous protective layer on the mild steel surface. This is supported by corrosion tests, which show that inhibition efficiency increases with both increasing concentration and immersion time.

Zeta potential is a fundamental parameter for understanding the colloidal stability of nanoparticles in a suspension. It represents the electric potential at the interface between the particle surface and the surrounding liquid medium, being directly related to the electrostatic repulsion between particles.^83^ Figure 16C shows the zeta potential values for the 50 and 400 ppm concentrations at *t*_0_. The zeta potential was +12.7 mV at a 50 ppm concentration and +6.9 mV at 400 ppm, suggesting weaker electrostatic repulsion between particles at higher concentrations, which favor agglomerate formation. This reduced repulsion at higher concentrations supports DLS observations in the acidic medium, where no free CQDs were detected at 400 ppm for *t*_0_. Given that the zeta potential is positive for both concentrations, it can be concluded that the aggregates are positively charged in an acidic solution.

The formation of positively charged agglomerates supports the hypothesis that the adsorption process is governed by physical adsorption, as corroborated by the varying temperature results, which show a higher activation energy in the presence of the inhibitor (64.3 kJ) compared with the uninhibited system (44.6 kJ). Thus, the protective film is primarily governed by electrostatic interactions between protonated functional groups in the CQDs (N, S, and O) and the negatively charged mild steel surface, which has been previously modified by chloride adsorption.

The formation of this film is essential to the inhibition effect observed in gravimetric and electrochemical tests. The increasing inhibition efficiency with concentration and exposure time observed in the gravimetric tests confirms that CQDs, by forming adsorbed agglomerate films, limit the access of aggressive ions (H^+^ and Cl^–^) to the metal surface.

Additionally, electrochemical tests indicate a mixed-type inhibitory behavior, with a predominant suppression of the anodic reaction, as evidenced by a shift of the corrosion potential to more positive values. This behavior indicates that CQD adsorption extends beyond electrostatic interactions, corroborating the XPS results, which revealed a chemical interaction between C and Fe (C–Fe) at 282.91 eV in the C 1s high-resolution spectrum, even at low intensity.

Altogether, these results show that the adsorption of garlic peel-derived CQDs is governed by a physisorption process, where the aggregation dynamics of the particles play a crucial role in the formation and stability of the protective layer. Therefore, both the concentration and exposure time are essential variables for determining the effectiveness of the inhibitor. A schematic of the proposed mechanism is shown in Figure 17.

**Figure 17:**
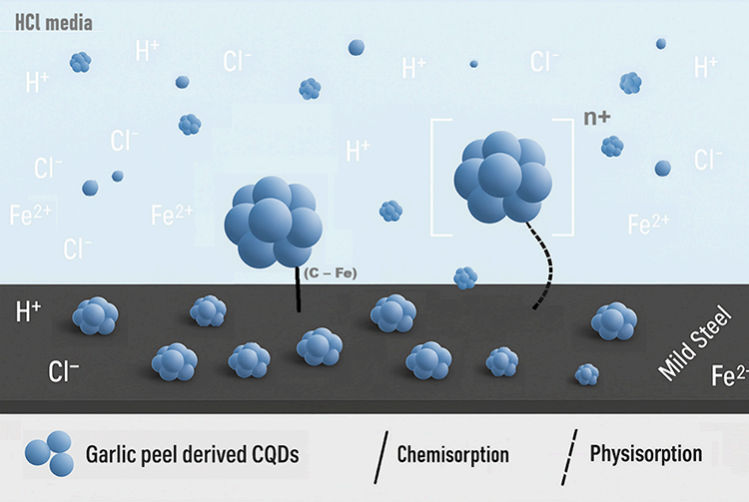
Schematic of the adsorption mechanism of garlic peel-derived CQDs on mild steel in 1 mol L^–1^ of HCl.

## 5 Conclusions

The TEM and DLS analyses confirmed the successful synthesis of carbon quantum dots (CQDs) from garlic peels with an average size of 2.95 ± 0.85 nm. FTIR analysis revealed chemical similarities between the CQDs and the garlic peel precursor, while XPS measurements indicated the presence of nitrogen and sulfur in the CQD structure, evidencing endogenous doping. The anticorrosive efficiency of the CQDs was validated through gravimetric, electrochemical, and surface characterization tests. Gravimetric studies demonstrated maximum inhibition efficiencies of 79.0 ± 0.36% at 300 ppm over 2 h and 95.8 ± 0.37% at 200 ppm over 24 h, indicating slow kinetics and sustained effectiveness. Temperature-dependent gravimetric tests suggested that the inhibition occurs primarily via a physical interaction. Electrochemical tests corroborated these findings, with inhibition efficiencies of 71.4 and 71.8% observed in electrochemical impedance spectroscopy and potentiodynamic polarization, respectively, at a concentration of 300 ppm. The polarization results classified the CQDs as mixed-type inhibitors with a predominant anodic effect. Surface characterization further confirmed these outcomes. SEM and AFM analyses showed reduced roughness and decreased iron dissolution on the MS surface in the presence of CQDs. Contact angle measurements indicated an increased hydrophobicity, supporting the formation of a protective film. XPS analysis of the mild steel surface verified the presence of CQDs and revealed evidence of chemical interaction between the metal and carbon, even at low intensity. Additionally, the XPS data, combined with an Arrhenius study, suggested that CQDs interact with the mild steel surface through both physical and chemical mechanisms, consistent with their complex structure and composition, as supported by isotherm studies. Finally, zeta potential and DLS analyses in the corrosive medium indicated that the inhibitory process is primarily due to the CQD aggregates.

These results demonstrate the potential of utilizing agro-industrial waste, such as garlic peel, as a starting material for synthesizing CQDs and their application as corrosion inhibitor. Thus, using waste as a value-added material would generate economic benefits for the agro-industrial sector and reduce environmental pollution that often occurs due to the burning and disposal of waste.

## References

[1] Mehta R. K., Gupta S. K., Yadav M. (2022). Studies on
pyrimidine derivative
as green corrosion inhibitor in acidic environment: Electrochemical
and computational approach. J. Environ. Chem.
Eng..

[2] Chigondo M., Chigondo F. (2016). Recent Natural Corrosion
Inhibitors for Mild Steel:
An Overview. J. Chem..

[3] Bobby
Kannan M., Rahuma M., Khakbaz H., Melchers R. (2022). Antipsychotic
drug waste: A potential corrosion inhibitor for mild steel in the
oil and gas industry. Waste Management..

[4] Saraswat V., Yadav M. (2021). Improved corrosion
resistant performance of mild steel under acid
environment by novel carbon dots as green corrosion inhibitor. Colloids Surf. A Physicochem Eng. Asp..

[5] Kadhim A. A., Al-Amiery A. A., Alazawi R., Al-Ghezi M. K. S., Abass R. H. (2021). Corrosion
inhibitors. A review. Int. J. Corrosion Scale
Inhibition.

[6] KM S., Praveen B. M., Devendra B. K. (2024). A review
on corrosion inhibitors:
Types, mechanisms, electrochemical analysis, corrosion rate and efficiency
of corrosion inhibitors on mild steel in an acidic environment. Results in Surfaces and Interfaces.

[7] Xu X., Wei H., Liu M., zhou L., Shen G., Li Q., Hussain G., Yang F., Fathi R., Chen H., Ostrikov K. (. (2021). Nitrogen-doped
carbon quantum dots for effective corrosion
inhibition of Q235 steel in concentrated sulphuric acid solution. Mater. Today Commun..

[8] Shi Y., Liu X., Wang M., Huang J., Jiang X., Pang J., Xu F., Zhang X. (2019). Synthesis of N-doped carbon quantum dots from bio-waste
lignin for selective irons detection and cellular imaging. Int. J. Biol. Macromol..

[9] Dhongde N. R., Das N. K., Banerjee T., Rajaraman P. V. (2024). Synthesis
of carbon quantum dots from rice husk for anti-corrosive coating applications:
Experimental and theoretical investigations. Ind. Crops Prod..

[10] Atchudan R., Jebakumar Immanuel Edison T. N., Shanmugam M., Perumal S., Somanathan T., Lee Y. R. (2021). Sustainable synthesis
of carbon quantum dots from banana peel waste using hydrothermal process
for in vivo bioimaging. Physica E Low Dimens
Syst. Nanostruct.

[11] Danial W. H., Abdullah M., Abu Bakar M. A., Yunos M. S., Ibrahim A. R., Iqbal A., Adnan N. N. (2022). The valorisation
of grass waste for
the green synthesis of graphene quantum dots for nonlinear optical
applications. Opt Mater. (Amst).

[12] Maestro C. A. R., de Sousa Malafaia A. M., Silva C. F., Nascimento C. S., Borges K. B., Simões T. A., Capelossi V. R., Bueno A. H. S. (2023). Corrosion resistance
improvement
of mild steel in different pH using peel garlic green inhibitor. Mater. Chem. Phys..

[13] Capanoglu E., Nemli E., Tomas-Barberan F. (2022). Novel Approaches
in the Valorization
of Agricultural Wastes and Their Applications. J. Agric. Food Chem..

[14] Feng L., Zheng S., Ma X., Zhu H., Hu Z., Sun Y. (2024). Enhancing corrosion protection in acidic environments through biomass-derived
carbon quantum dots. Microchemical Journal..

[15] Zheng S., Feng L., Hu Z., Li J., Zhu H., Ma X. (2023). Study on the corrosion inhibition
of biomass carbon quantum dot self-
aggregation on Q235 steel in hydrochloric acid. Arabian Journal of Chemistry..

[16] Long W. J., Li X. Q., Yu Y., He C. (2022). Green synthesis
of
biomass-derived carbon dots as an efficient corrosion inhibitor. J. Mol. Liq..

[17] Kamaruzzaman W.M.I.W.M., Shaifudin M. S., Nasir N. A. M., Badruddin M. A., Yusof N., Adnan A., Aziz N. A., Nik W. M. N. W., Haque J., Murmu M., Banerjee P., Mohd Ghazali M. S. (2024). Experimental,
DFT and molecular dynamic simulation of Andrographis paniculata as
corrosion inhibitor for mild steel in artificial seawater. Mater. Chem. Phys..

[18] Verma C., Chauhan D. S., Aslam R., Banerjee P., Aslam J., Quadri T. W., Zehra S., Verma D. K., Quraishi M. A., Dubey S., AlFantazi A., Rasheed T. (2024). Principles and theories
of green chemistry for corrosion science and engineering: design and
application. Green Chemistry..

[19] Zamindar S., Mandal S., Murmu M., Banerjee P. (2024). Unveiling the future
of steel corrosion inhibition: a revolutionary sustainable odyssey
with a special emphasis on N+-containing ionic liquids through cutting-edge
innovations. Mater. Adv..

[20] Liu S., Dong K., Guo F., Qiao Q., Xu L., Wang J., Kong L., Zhang Y., Chang J., Yan W. (2023). Green synthesis of
nitrogen self-doped porous carbons from waste
garlic peels for high-performance supercapacitor applications. J. Anal Appl. Pyrolysis..

[21] Oliveira J. T. d., Oliveira R. A. d., Furtado
Junior M. R. (2021). CONTRIBUTION
OF SOIL ATTRIBUTES AND MORPHOLOGICAL VARIABLES TO YIELD OF IRRIGATED
GARLIC. Engenharia Agrícola..

[22] Vargas J., Tarnonsky F., Maderal A., Fernandez-Marenchino I., Podversich F., Cuervo W., Gomez-Lopez C., Schulmeister T., DiLorenzo N. (2023). Effects of Processing Methods and
Inclusion Levels of Dried Garlic on In Vitro Fermentation and Methane
Production in a Corn Silage-Based Substrate. Animals.

[23] Qian B., Hou B., Zheng M. (2013). The inhibition
effect of tannic acid on mild steel
corrosion in seawater wet/dry cyclic conditions. Corros. Sci..

[24] da
Cunha J. N., Evangelista B. D. V., Xavier A. V., da Silva T. U., de Oliveira S. M., de Araújo J. R., Archanjo B. S., de Paula
Machado S., Rezende M. J. C., das Chagas Almeida T., Mattos O. R., D’Elia E. (2023). Study of furfural derivatives as
a possible green corrosion inhibitor for mild steel in CO2-saturated
formation water. Corros. Sci..

[25] Carlos M. P., Xavier N. F., da Silva A. M., Neves M. A., Echevarria A., Bauerfeldt G. F. (2021). Synergy
between Experimental and Theoretical Investigations
Reveals the AntiCorrosion Efficiency of Imine-Chalcones. J. Braz Chem. Soc..

[26] Setianto S., Men L. K., Bahtiar A., Panatarani C., Joni I. M. (2024). Carbon quantum dots with honeycomb structure: a novel
synthesis approach utilizing cigarette smoke precursors. Sci. Rep..

[27] Smith R. J., Horgan B. H. N. (2021). Nanoscale Variations in Natural Amorphous
and Nanocrystalline
Weathering Products in Mafic to Intermediate Volcanic Terrains on
Earth: Implications for Amorphous Detections on Mars. J. Geophys Res. Planets.

[28] Dong Y., Shao J., Chen C., Li H., Wang R., Chi Y., Lin X., Chen G. (2012). Blue luminescent graphene quantum
dots and graphene oxide prepared by tuning the carbonization degree
of citric acid. Carbon N Y..

[29] Liu S. S., Wang C. F., Li C. X., Wang J., Mao L. H., Chen S. (2014). Hair-derived carbon
dots toward versatile multidimensional fluorescent
materials. J. Mater. Chem. C Mater..

[30] Roy P., Chen P. C., Periasamy A. P., Chen Y. N., Chang H. T. (2015). Photoluminescent
carbon nanodots: synthesis, physicochemical properties and analytical
applications. Materials Today..

[31] Li H., Sun C., Vijayaraghavan R., Zhou F., Zhang X., MacFarlane D. R. (2016). Long lifetime
photoluminescence in N, S co-doped carbon
quantum dots from an ionic liquid and their applications in ultrasensitive
detection of pesticides. Carbon N Y..

[32] Liu S., Tian J., Wang L., Zhang Y., Qin X., Luo Y., Asiri A. M., Al-Youbi A. O., Sun X. (2012). Hydrothermal treatment
of grass: A low-cost, green route to nitrogen-doped, carbon-rich,
photoluminescent polymer nanodots as an effective fluorescent sensing
platform for label-free detection of Cu­(II) ions. Adv. Mater..

[33] Zhu A., Qu Q., Shao X., Kong B., Tian Y. (2012). Carbon-dot-based dual-emission
nanohybrid produces a ratiometric fluorescent sensor for in vivo imaging
of cellular copper ions. Angewandte Chemie -
International Edition..

[34] Jia J., Sun Y., Zhang Y., Liu Q., Cao J., Huang G., Xing B., Zhang C., Zhang L., Cao Y. (2020). Facile and
Efficient Fabrication of Bandgap Tunable Carbon Quantum Dots Derived
From Anthracite and Their Photoluminescence Properties. Front Chem..

[35] Tammina S. K., Yang D., Koppala S., Cheng C., Yang Y. (2019). Highly photoluminescent
N, P doped carbon quantum dots as a fluorescent sensor for the detection
of dopamine and temperature. J. Photochem. Photobiol.
B.

[36] Dai F., Zhuang Q., Huang G., Deng H., Zhang X. (2023). Infrared Spectrum
Characteristics and Quantification of OH Groups in Coal. ACS Omega..

[37] Ahmed H. T., Abdullah O. G. (2020). Structural and ionic conductivity
characterization
of PEO:MC-NH4I proton-conducting polymer blend electrolytes based
films. Results Phys..

[38] Kurdekar A., Chunduri L. A. A., Bulagonda E. P., Haleyurgirisetty M. K., Kamisetti V., Hewlett I. K. (2016). Comparative performance
evaluation
of carbon dot-based paper immunoassay on Whatman filter paper and
nitrocellulose paper in the detection of HIV infection. Microfluid Nanofluidics.

[39] Zhang S. R., Cai S. K., Wang G. Q., Cui J. Z., Gao C. Z. (2021). One-step
synthesis of N, P-doped carbon quantum dots for selective and sensitive
detection of Fe2+ and Fe3+ and scale inhibition. J. Mol. Struct..

[40] Zhao L., Wang Y., Zhao X., Deng Y., Xia Y. (2019). Facile Synthesis
of Nitrogen-Doped Carbon Quantum Dots with Chitosan for Fluorescent
Detection of Fe3+. Polymers.

[41] Barbe A., Jouve P. (1971). Force constants and form of vibration of sulfur dioxide from infrared
spectrum of S18O2. J. Mol. Spectrosc..

[42] Hemmati A., Emadi H., Nabavi S. R. (2023). Green Synthesis
of Sulfur- and Nitrogen-Doped
Carbon Quantum Dots for Determination of L-DOPA Using Fluorescence
Spectroscopy and a Smartphone-Based Fluorimeter. ACS Omega..

[43] Wang C., Shi H., Yang M., Yao Z., Zhang B., Liu E., Hu X., Xue W., Fan J. (2021). Biocompatible sulfur nitrogen co-doped
carbon quantum dots for highly sensitive and selective detection of
dopamine. Colloids Surf. B Biointerfaces..

[44] Riaz S., Park S. J. (2022). Thioacetamide-derived
nitrogen and sulfur co-doped
carbon quantum dots for “green” quantum dot solar cells. Journal of Industrial and Engineering Chemistry..

[45] Tripathi K. M., Ahn H. T., Chung M., Le X. A., Saini D., Bhati A., Sonkar S. K., Kim M. Il, Kim T. Y. (2020). N, S, and
P-Co-doped Carbon Quantum Dots: Intrinsic Peroxidase Activity in a
Wide pH Range and Its Antibacterial Applications. ACS Biomater Sci. Eng..

[46] Zhu M., Guo L., He Z., Marzouki R., Zhang R., Berdimurodov E. (2022). Insights into
the newly synthesized N-doped carbon dots for Q235 steel corrosion
retardation in acidizing media: A detailed multidimensional study. J. Colloid Interface Sci..

[47] Yang Y., Lu R., Chen W., Mei P., Lai L. (2022). Amphiphilic carbon
dots as high-efficiency corrosion inhibitor for N80 steel in HCl solution:
Performance and mechanism investigation. Colloids
Surf. A Physicochem Eng. Asp.

[48] Abd-El-Nabey B. A., Mahmoud M. E., Abdelrahman A., Abd-El-Fatah M. A. (2024). Effective
Performance of Derived Sustainable Lupine Carbon Quantum Dots as a
Superior Inhibitor Material for Carbon Steel Corrosion in a Hydrochloric
Acidic Environment (1.0 Molar). Ind. Eng. Chem.
Res..

[49] Nunes R. da S., Magno Paiva V., de Oliveira S. M., da Silva de Almeida C. M., de Oliveira M. S., de Araujo J. R., Archanjo B. S., Suguihiro N. M., D’Elia E. (2024). Sugar Cane (Saccharum officinarum L.) Waste Synthesized
Si,N,S-Carbon Quantum Dots as High-Performance Corrosion Inhibitors
for Mild Steel in Hydrochloric Acid. ACS Omega..

[50] Magno
Paiva V., Massafra de Oliveira S., Muniz da
Silva de Almeida C., Rodrigues de Araujo J., Soares Archanjo B., D'Elia E. (2024). Pumpkin (Cucurbita maxima) seed-derived nitrogen, phosphorus,
and sulfur carbon quantum dot as an inhibitor of corrosion for mild
steel in HCl solution. J. Mater. Res. Technol..

[51] Alharthi N. H., El-Hashemy M. A., Derafa W. M., Althobaiti I. O., Altaleb H. A. (2022). Corrosion inhibition
of mild steel by highly stable
polydentate schiff base derived from 1,3- propanediamine in aqueous
acidic solution. Journal of Saudi Chemical Society..

[52] Murphy O. P., Vashishtha M., Palanisamy P., Kumar K. V. (2023). A Review on the
Adsorption Isotherms and Design Calculations for the Optimization
of Adsorbent Mass and Contact Time. ACS Omega.

[53] Sanni O., Popoola A. P. I., Fayomi O. S. I. (2019). Temperature Effect, Activation Energies
and Adsorption Studies of Waste Material as Stainless Steel Corrosion
Inhibitor in Sulphuric Acid 0.5 M. J. Bio Tribocorros.

[54] Egbosiuba T. C., Chukwunyere I. E., Awere C. O., Amadi N. M., Okafor B. O., Ezeugo J. O., Onukwuli O. D., Arrousse N., Berdimurodov E., Anadebe V. C. (2025). Psidium guajava L. extract as corrosion inhibitor for
mild steel in an acidic environment: Experimental and computational
insights. Int. J. Electrochem Sci..

[55] Ji H., Zhao X., Qiao Z., Jung J., Zhu Y., Lu Y., Zhang L. L., MacDonald A. H., Ruoff R. S. (2014). Capacitance of carbon-based
electrical double-layer capacitors. Nat. Commun..

[56] Li Y., Zhang S., Ding Q., Qin B., Hu L. (2019). Versatile
4, 6-dimethyl-2-mercaptopyrimidine based ionic liquids as high-performance
corrosion inhibitors and lubricants. J. Mol.
Liq..

[57] Al-Amiery A. A., Ahmed M. H. O., Abdullah T. A., Gaaz T. S., Kadhum A. A. H. (2018). Electrochemical
studies of novel corrosion inhibitor for mild steel in 1 M hydrochloric
acid. Results Phys..

[58] Wang F., Ma T., Zhang S., Tan B., Guo L., Du H., Wang X., Han X., Liu R. (2024). Corrosion inhibition
performance and mechanism of nitrogen-containing organic compounds
on copper in an alkaline slurry. J. Mol. Liq..

[59] Idígoras J., Guillén E., Ramos F. J., Anta J. A., Nazeeruddin M. K., Ahmad S. (2014). Highly efficient flexible cathodes for dye sensitized solar cells
to complement Pt@TCO coatings. J. Mater. Chem.
A Mater..

[60] Wu J. (2022). Understanding
the Electric Double-Layer Structure, Capacitance, and Charging Dynamics. Chem. Rev..

[61] Abdel-Rehim S. S., Khaled K. F., Al-Mobarak N. A. (2011). Corrosion
inhibition of iron in hydrochloric
acid using pyrazole. Arabian Journal of Chemistry..

[62] Marhamati F., Mahdavian M., Bazgir S. (2021). Corrosion mitigation of mild steel
in hydrochloric acid solution using grape seed extract. Sci. Rep.

[63] Alharbi M., Aslam R., Khan A., Alamry K. A., Al-Hadeethi Y., Bekyarova E., Alqahtani S., Hussein M. A. (2025). Insight into the
time-dependent corrosion protection of mild steel by green ionic liquids:
a combined electrochemical, surface, and computational study. RSC Adv..

[64] Rathod M. R., Rajappa S. K. (2022). Corrosion inhibition effect of Cycas revoluta leaves
extract on corrosion of soft-cast steel in hydrochloric acid medium. Electrochem. Sci. Adv..

[65] Abdellaoui O., Skalli M. K., Haoudi A., Rodi Y. K., Mazzah A., Arrousse N., Taleb M., Ghibate R., Senhaji O. (2021). Study of the
inhibition effect of a new cationic surfactant on mild steel corrosion
in a 1 M HCl solution. Mater. Today Proc..

[66] Barros I. B. d., Kappel M. A. A., Santos P. M. d., Veiga Junior V. F. d., D'Elia E., Bastos I. N. (2016). The inhibitory action of *Bauhinia purpurea* extracts on the corrosion of carbon steel
in sulfuric acid medium. Mater. Res..

[67] Souza F. S. d., Gonçalves R. S., Spinelli A. (2013). Assessment of caffeine
adsorption onto mild steel surface as an eco-friendly corrosion inhibitor. J. Braz Chem. Soc..

[68] Sudhakaran R., Deepa T., Babu S., Mohan S. (2023). New developed for generating
superhydrophobic surface modification on mild steel for corrosion
protection. Results Chem..

[69] Cheirmakani B. M., M K., S B., RB J. R. (2024). Development of superhydrophobic
surface
on mild steel by a facile approach and analyzing its self-cleaning
and anti-freezing properties. Results in Surfaces
and Interfaces.

[70] Kriaa A., Hamdi N., Jbali K., Tzinmann M. (2008). Kinetics study
of iron
dissolution in highly concentrated acidic media using Hammett acidity
function. Corros. Sci..

[71] Hou Z., Yan P., Sun B., Elshekh H., Yan B. (2019). An excellent soft magnetic
Fe/Fe3O4-FeSiAl composite with high permeability and low core loss. Results Phys..

[72] Saraswat V., Kumari R., Yadav M. (2022). Novel carbon dots as
efficient green
corrosion inhibitor for mild steel in HCl solution: Electrochemical,
gravimetric and XPS studies. Journal of Physics
and Chemistry of Solids..

[73] Makrides A. C. (1960). Dissolution
of Iron in Sulfuric Acid and Ferric Sulfate Solutions. J. Electrochem. Soc..

[74] Ouakki M., Aribou Z., El Hajri F., Ech-chihbi E., Benzekri Z., Lachhab R., Srhir B., Patrick M., Almeer R., Galai M., Boukhris S., Cherkaoui M. (2024). A study on
the corrosion inhibition impact of newly synthesized quinazoline derivatives
on mild steel in 1.0 M HCl: Experimental, surface morphological (SEM-EDS
and FTIR) and computational analysis. Int. J.
Electrochem Sci..

[75] Furlan A., Jansson U., Lu J., Hultman L., Magnuson M. (2015). Structure
and bonding in amorphous iron carbide thin films. Journal of Physics: Condensed Matter..

[76] Wang C., Sun D., Zhuo K., Zhang H., Wang J. (2014). Simple and green synthesis
of nitrogen-, sulfur-, and phosphorus-co-doped carbon dots with tunable
luminescence properties and sensing application. RSC Adv..

[77] Wang Y., Carraro G., Dawczak-Dębicki H., Synoradzki K., Savio L., Lewandowski M. (2020). Reversible
and irreversible structural
changes in FeO/Ru(0 0 0 1) model catalyst subjected to atomic oxygen. Appl. Surf. Sci..

[78] El-Haddad M. A. M., Bahgat Radwan A., Sliem M. H., Hassan W. M. I., Abdullah A. M. (2019). Highly efficient eco-friendly corrosion inhibitor for
mild steel in 5 M HCl at elevated temperatures: experimental &
molecular dynamics study. Sci. Rep..

[79] Koushik D., Verhees W. J. H., Zhang D., Kuang Y., Veenstra S., Creatore M., Schropp R. E. I. (2017). Atomic
layer deposition enabled perovskite/PEDOT
solar cells in a regular n-i-p architectural design. Adv. Mater. Interfaces..

[80] Zhang W. (2014). Nanoparticle
Aggregation: Principles and Modeling. Adv. Exp.
Med. Biol..

[81] Hotze E. M., Phenrat T., Lowry G. V. (2010). Nanoparticle Aggregation: Challenges
to Understanding Transport and Reactivity in the Environment. J. Environ. Qual..

[82] Medina-Lopez D., Liu T., Osella S., Levy-Falk H., Rolland N., Elias C., Huber G., Ticku P., Rondin L., Jousselme B., Beljonne D., Lauret J. S., Campidelli S. (2023). Interplay
of structure and photophysics of individualized rod-shaped graphene
quantum dots with up to 132 sp^2^ carbon atoms. Nat. Commun..

[83] Pochapski D. J., Carvalho dos Santos C., Leite G. W., Pulcinelli S. H., Santilli C. V. (2021). Zeta Potential and Colloidal Stability Predictions
for Inorganic Nanoparticle Dispersions: Effects of Experimental Conditions
and Electrokinetic Models on the Interpretation of Results. Langmuir..

